# The surface and micellar properties of ethanolamine based surface active ionic liquids in the presence of drug aspirin

**DOI:** 10.1038/s41598-025-20946-2

**Published:** 2025-10-22

**Authors:** Elaheh Janbezar, Hemayat Shekaari, Shima Ghasemzadeh, Mohammad Bagheri Hokm Abad

**Affiliations:** Department of Physical Chemistry, Faculty of Chemistry, University of Tabriz, Tabriz, 5166616471 Iran

**Keywords:** Surface tension, Electrical conductivity, Biobased surface-active ionic liquids, COSMO analysis, Aspirin, Biochemistry, Chemistry, Physical chemistry

## Abstract

Bio-based surface-active ionic liquids (SAILs) offer promising advantages for pharmaceutical applications, particularly in enhancing drug solubility and bioavailability. Aspirin, classified under the Biopharmaceutics Classification System (BCS) as poorly soluble in the gastrointestinal tract, require effective solubilization strategies for improved therapeutic efficacy. This study investigates the micellization behavior and thermophysical properties of three natural based SAILs (2-hydroxyethyl)ammonium oleate ([2-HEA][Ole]), bis(2-hydroxyethyl)ammonium oleate ([BHEA][Ole]), and tris(2-hydroxyethyl)ammonium oleate ([THEA][Ole]) in aqueous solutions of aspirin at 298 K. Micellization characteristics, including critical micelle concentration (CMC), were determined using electrical conductivity and surface tension measurements for SAILs in the presence of the aspirin aqueous solutions systems at 298 K. Key interfacial parameters such as interface surface pressure ($\Pi$), minimum surface area occupied per molecule (${A_{\hbox{min} }}$), Gibbs maximum excess surface concentration (${\Gamma _{\hbox{max} }}$) were also calculated. Additionally, the Conductor-like Screening Model (COSMO) was employed to elucidate molecular interactions between SAILs and the studied drug. The results indicate that CMC values decrease in the presence of aspirin. Among the studied systems, [THEA][Ole] exhibited the lowest CMC, as determined by electrical conductivity and surface tension measurements, particularly in the presence of higher concentrations of aspirin in aqueous media. Furthermore, COSMO analysis revealed that [THEA][Ole], possessing the highest surface cavity volume (*V*), demonstrated the most favorable interactions with aspirin, highlighting its potential as an effective solubilizing agent. Finally, interactions between SAILs and aspirin were investigated through limiting molar conductivity $\wedge_0$ , and association constant $K_A$ , determination.

**Supplementary Information:**

The online version contains supplementary material available at 10.1038/s41598-025-20946-2.

## Introduction

Nonsteroidal anti-inflammatory drugs (NSAIDs), including aspirin, are widely used for their analgesic, anti-inflammatory, and antipyretic effects. However, their high permeability combined with poor aqueous solubility significantly limits their bioavailability^1^. This challenge highlights the necessity for innovative formulation strategies aimed at enhancing dissolution rates and therapeutic efficacy. The pharmaceutical industry has made numerous attempts to address these issues through various physical and chemical modifications, as well as other methods^2^. The physical modification strategy primarily focuses on reducing particle size using techniques such as micronization and nanosuspension. It also involves altering the crystal habit, which includes developing polymorphs, forming amorphous forms, and employing crystallization processes^3^. Additionally, this approach encompasses dispersing drugs within different carriers, which may consist of eutectic mixtures, solid dispersions, solid solutions, and the application of cryogenic techniques^4^.

The utilization of the chemical method is one of the most commonly used approaches for enhancing the drug-related properties of pharmaceutical compounds. This method enhances drug properties by applying changes within the change of pH, uses buffers, etc. Among the indicated methods, the micellization method in aqueous environments is potentially the most promising for achieving the desired purpose^5^. These micelles, which feature hydrophobic cores and hydrophilic exteriors, can encapsulate hydrophobic drugs, thereby improving solubility and dissolution rates. Several factors influence the effectiveness of this process, including surfactant nature and concentration, charge, and hydrophilic-lipophilic balance^6^. Traditional surfactants, such as sodium lauryl sulfate (SLS) and cetyltrimethylammonium bromide (CTAB), are well-known for the anionic and cationic nature of their head groups, respectively. However, despite their remarkable characteristics, these substances lack an important criterion related to their tunability aspects^7^.

The surface-active ionic liquids (SAILs) have garnered considerable attention within the realm of pharmaceutical formulations due to their unique tunable surface activity and superior physicochemical properties^8^. These compounds possess the ability to self-assemble into micelles when in aqueous solutions, a process that is significantly influenced by the structural characteristics of their constituent cations and anions. The hydroxyl functionalization in the studied SAILs ([2-HEA][Ole], [BHEA][Ole], [THEA][Ole]) enhances intermolecular interactions, critical for micelle stability and drug solubilization^9^. The presence of hydroxyl groups not only facilitates stronger intermolecular interactions but also contributes to the stability and functionality of the micelles formed^10^. The implications of these properties are profound, as they can lead to improved drug solubility, bioavailability, and targeted delivery in pharmaceutical applications. Understanding the micellization behavior and the influence of ionic liquid structure on their performance is essential for the development of more effective pharmaceutical formulations^11^.

Electrical conductivity is a fundamental characteristic that plays a pivotal role in the utilization of ionic liquids (ILs) across various domains, including energy storage, drug delivery, and biomedical applications. The introduction of hydroxyl groups into ammonium oleate based ILs is expected to improve their electrical conductivity by affecting the complex interactions among viscosity, ion mobility, and intermolecular forces. While previous research has investigated the thermophysical properties of similar ionic liquids, there remains a significant gap in the literature specifically focused on the electrical conductivity of hydroxyl-functionalized ammonium oleate ILs^12^. This gap highlights the need for targeted studies to elucidate the mechanisms by which hydroxyl groups influence conductivity. Moreover, the interaction between these ionic liquids and non-steroidal anti-inflammatory drugs (NSAIDs), such as aspirin, offers a compelling area for further exploration. The limited aqueous solubility of NSAIDs, combined with their ionizable functional groups, suggests that their incorporation into ILs could substantially alter the electrochemical properties of these solvents. This alteration may impact critical factors such as solubility, dissociation, and ion transport dynamics, thereby influencing the overall efficacy of drug delivery systems that utilize ionic liquids^13^.

**Fig. 1:**
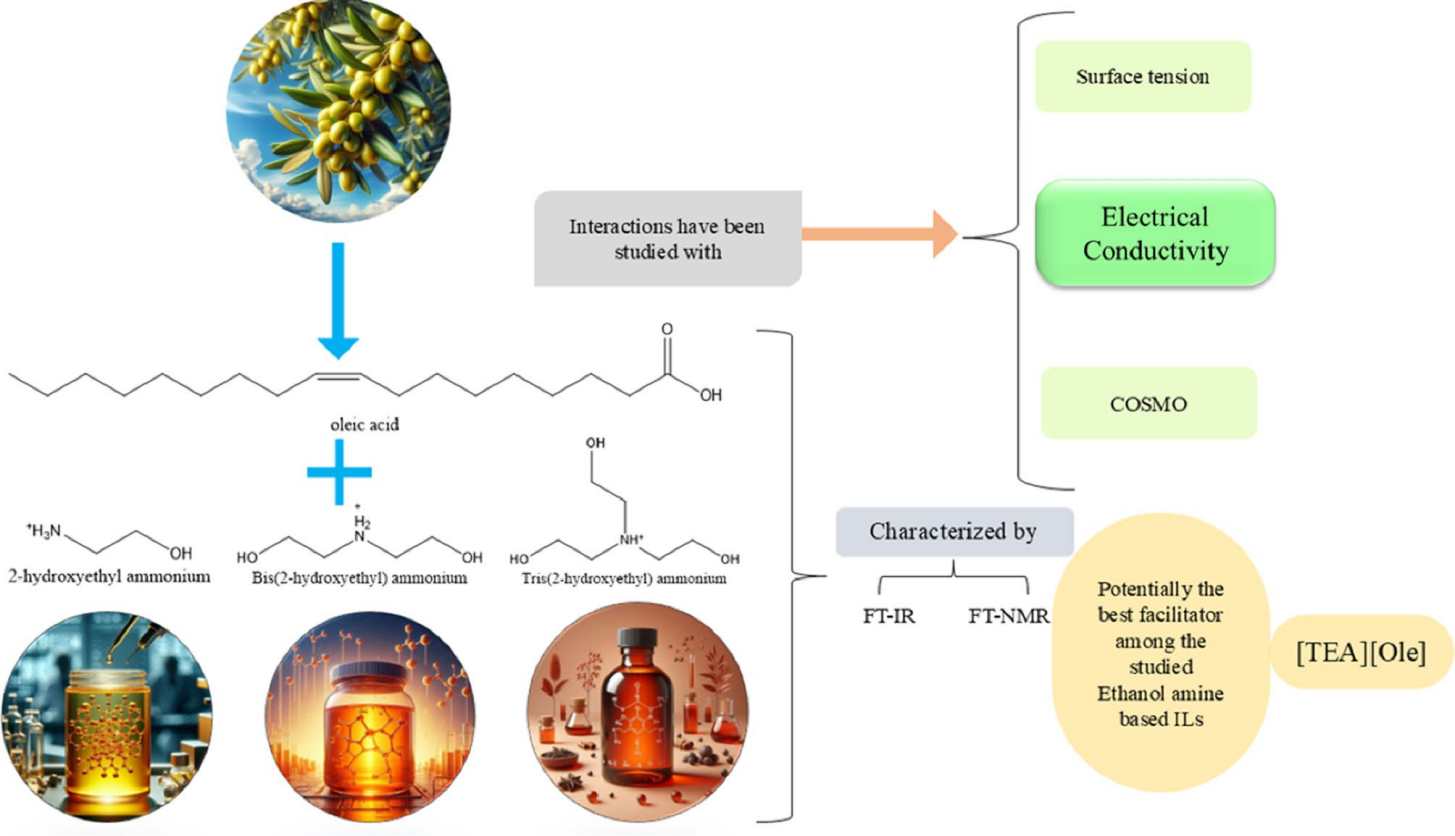
Novel hydroxyethylammonium-based surface-active ionic liquids revealing drug aspirin interactions through thermophysical and COSMO methods.

This study employs electrical conductivity and static surface tension measurements at 298 K and ambient pressure to assess the critical micelle concentration (CMC) and limiting molar conductivity (*Λ*_0_) of SAILs in the presence of aqueous aspirin solutions. Additionally, COSMO analysis is utilized to gain insights into the intermolecular interactions between aspirin and SAIL molecules in aqueous media. The resulting data on surface tension and electrical conductivity provide valuable information about ion mobility and interfacial behavior associated with micelle formation. A range of aqueous aspirin solutions containing various concentrations of SAILs were prepared for the measurement of surface tension and electrical conductivity. A static force tensiometer utilizing the Wilhelmy plate method was employed to ensure high accuracy and reliability in surface tension measurements. These measurements facilitated the calculation of surface-related properties, including CMC, Gibbs free energy of micellization (*∆G*_mic_), and the minimum surface area per surfactant molecule (*A*_min_). Interfacial electron density was analyzed through density functional theory (DFT) calculations, employing the COSMO model provided by Dmol^3^. This analysis determined the surface and total area of the cavity (*A*), cavity volume (*V*), dielectric solvation energy, and the highest and lowest unoccupied molecular orbitals (HOMO and LUMO), contributing to a comprehensive understanding of the electrostatic distribution and molecular structure. Furthermore, this study investigates the micellization behavior of hydroxyl-functionalized ammonium oleate SAILs, specifically (2-hydroxyethyl)ammonium oleate, bis(2-hydroxyethyl)ammonium oleate, and tris(2-hydroxyethyl)ammonium oleate, alongside NSAIDs like aspirin. A combination of experimental and computational techniques will be employed to explore micellization and solubilization efficiencies. The findings of this research will contribute to a deeper understanding of the role of SAILs in enhancing the drug related properties by highlighting their potential in biomedical applications. Given the increasing significance of SAILs in pharmaceuticals, a thorough understanding of the factors influencing their electrical conductivity is essential for optimizing performance. Additionally, an overview of the novelty of the utilized work has been presented within the Fig. 1.

## Materials and methods

### Chemicals

A comprehensive description of the utilized the chemicals has been provided in Table 1. This includes the chemical name, chemical formula, origin, CAS registry number (CAS no.), molar mass, and mass fraction (purity) of the chemicals employed in this study. The deionized-double distilled water used in this research had a specific conductance of less than 1 µS·cm^−1^.

**Table 1:** The specification of the utilized chemicals ^a^.

Chemical name	Chemical Formula	Provenance (Origin)	CAS.no	Molar mass (g mol^−1^)	Mass fraction (purity)
2-Hydroxyethylamine or monoethanolamine	C_2_H_7_NO	Merck	141-43-5	61.08	> 99%
Bis(2-hydroxyethyl)amine or diethanolamine	C_4_H_11_NO_2_	Merck	111-42-2	105.14	> 99%
Tris(2-hydroxyethyl)amine or triethanolamine	C_6_H_15_NO_3_	Merck	102-71-6	149.19	> 99%
Oleic acid	C_18_H_34_O_2_	Merck	112-80-1	318.13	> 99%
Aspirin	C_9_H_8_O_4_	Merck	50-78-2	180.16	> 99%
(2-Hydroxyethyl)ammonium oleate [2-HEA][Ole]	C_20_H_41_NO_3_	Synthesized	-	343.55	> 98%
Bis(2-hydroxyethyl)ammonium oleate [BHEA][Ole]	C_22_H_45_NO_4_	Synthesized	-	387.33	> 98%
Tris(2-hydroxyethyl)ammonium oleate [THEA][Ole]	C_24_H_49_NO_5_	Synthesized	-	431.66	> 98%

^a^ All of the attained chemicals from the supplier were used without further purification.

### Synthesis process of the SAILs

The synthesized surface active ionic liquids (SAILs) include (2-hydroxyethyl)ammonium oleate ([2-HEA][Ole]), bis(2-hydroxyethyl)ammonium oleate ([BHEA][Ole]), and tris(2-hydroxyethyl)ammonium oleate ([THEA][Ole]). These compounds were produced through a careful acid-base neutralization reaction, which involved the combination of oleic acid with three different ethanolamine derivatives: monoethanolamine (MEA), diethanolamine (DEA), and triethanolamine (TEA). Each component, oleic acid and ethanolamine, was utilized in a 1:1 mol ratio^14^. The schematic of the synthesis route of the studied SAILs has been provided in Fig S1.

Initially, oleic acid was introduced into a reaction vessel and heated to a temperature range of (323 to 333) K while being continuously stirred to achieve a homogeneous liquid state. Following this initial step, the corresponding ethanolamine was added incrementally, with constant stirring maintained throughout the process. The characteristics of the final product were significantly influenced by the specific ethanolamine utilized: the application of monoethanolamine (MEA) resulted in a liquid with moderate viscosity, diethanolamine (DEA) produced a more viscous liquid with enhanced surface-active properties, and triethanolamine (TEA) yielded a product with pronounced emulsifying capabilities. The neutralization reaction was characterized as exothermic, necessitating meticulous temperature regulation to prevent overheating, with optimal temperature maintenance below 343 K. Throughout the reaction, several observable changes were noted, including a color transition from yellow to light brown and a significant increase in viscosity. Following the complete addition of ethanolamine, the mixture was agitated at a temperature range of (333–343) K for a duration of (2 to 4) h to ensure the reaction reached completion^15^. Subsequent to this reaction phase, the resultant product underwent vacuum drying and was subjected to heating at temperatures between (313 and 323) K to remove any residual solvents. The purity of the synthesized SAILs has been investigated by the FT-IR and FT-NMR spectroscopies (presented in Figs S2–S7 respectively).

**Fig. 2:**
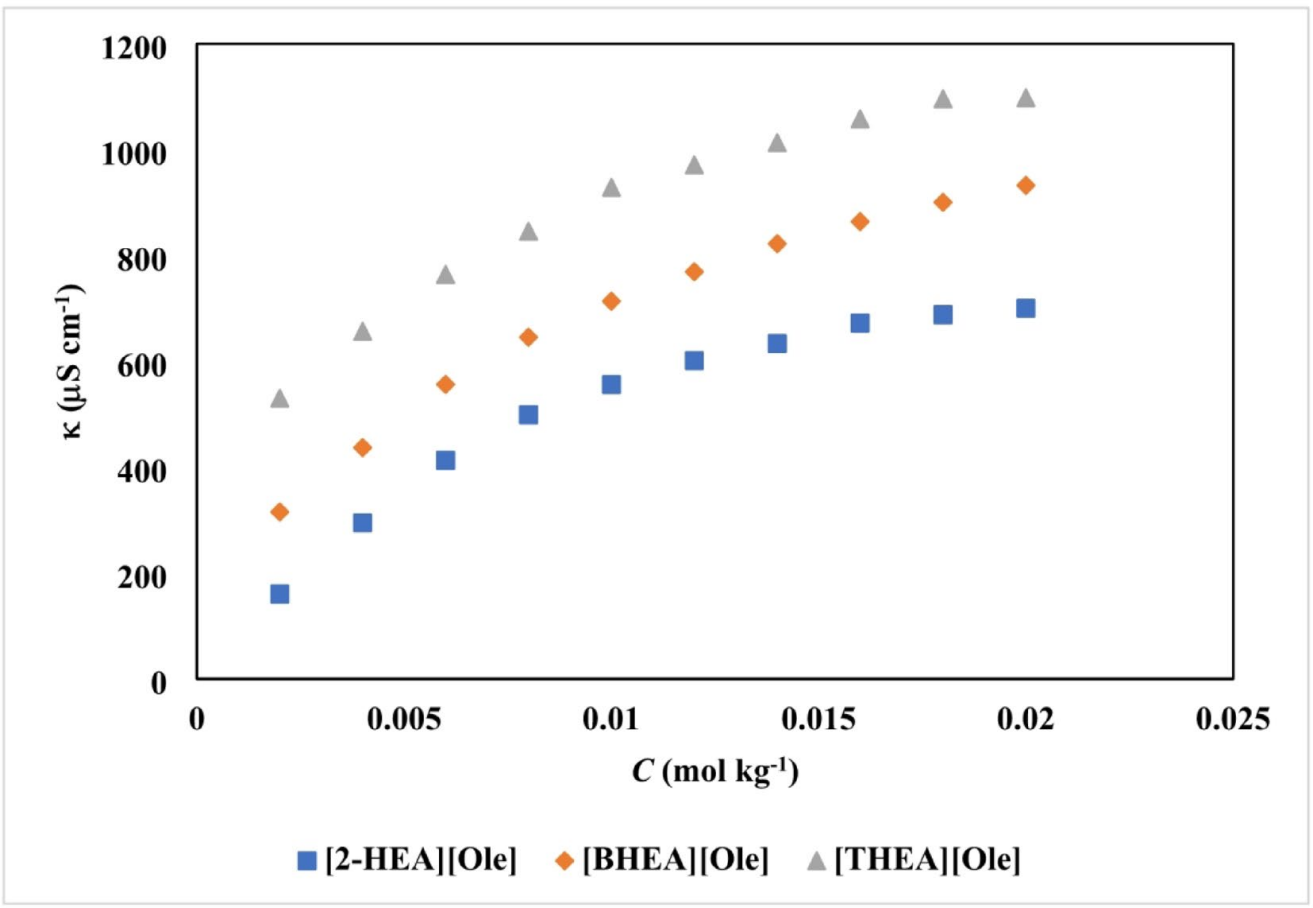
Specific conductivity (*κ*) of SAILs in a fixed concentration of aqueous aspirin solutions (0.0300 mol·kg^-1^) at 298 K.

**Fig. 3:**
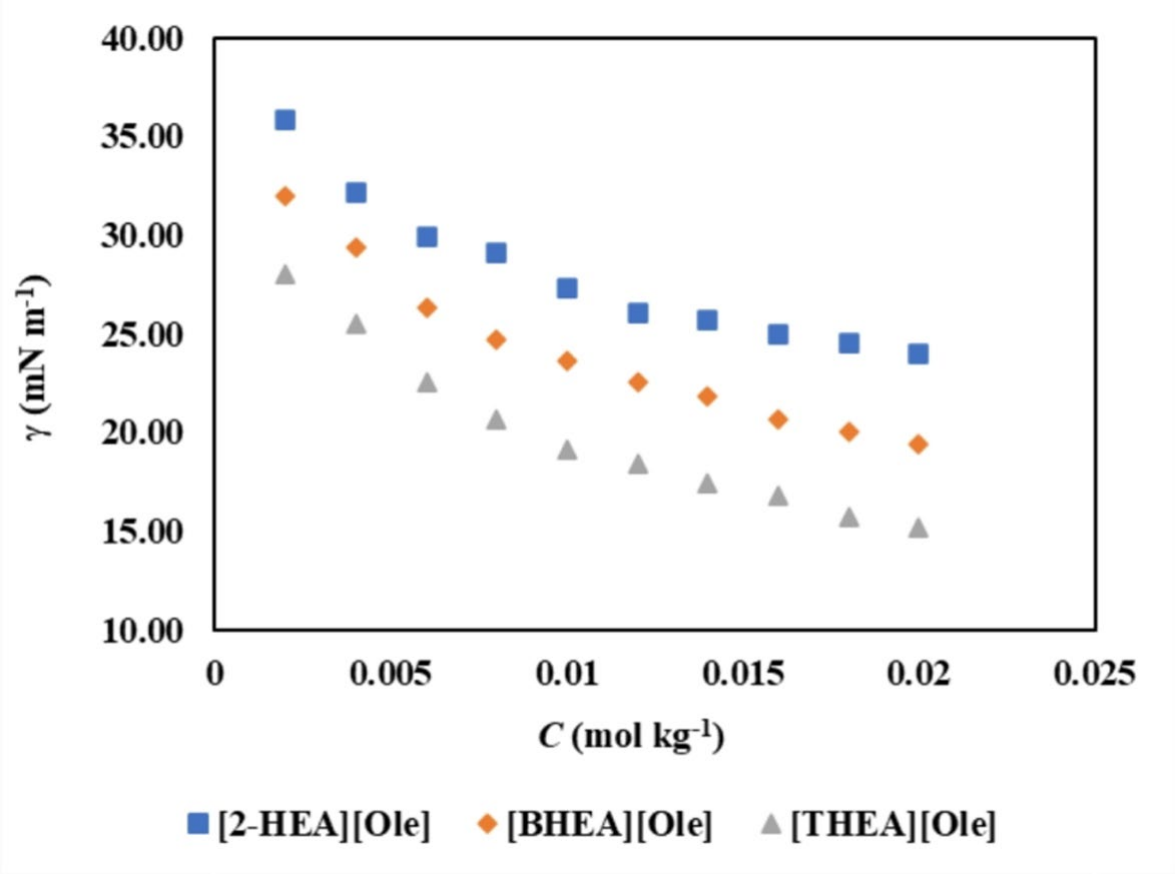
Surface tension (*γ*) of studied SAILs in aqueous aspirin solutions with 0.0100 molality concentrations of aspirin (mol·kg^−1^) at 298 K.

**Fig. 4:**
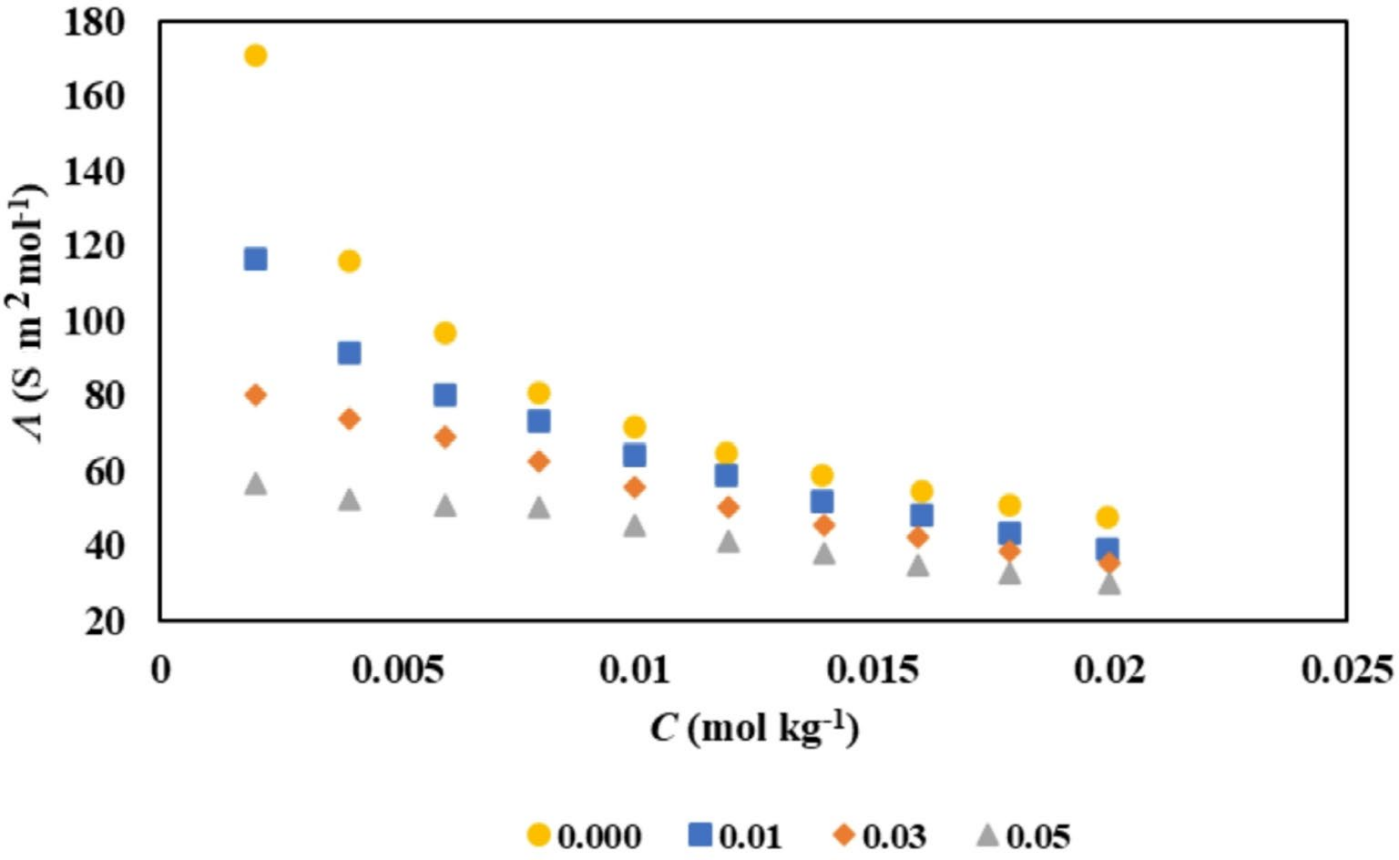
Molar conductivities (*Λ*) of [2-HEA][Ole] in aqueous aspirin solution with different molalities of aspirin (⬤ 0.0000 mol kg^-1^, ■ 0.0010 mol kg^−1^, ♦ 0.0300 mol kg^−1^, ▲ 0.0500 mol kg^−1^) at 298 K.

**Fig. 5:**
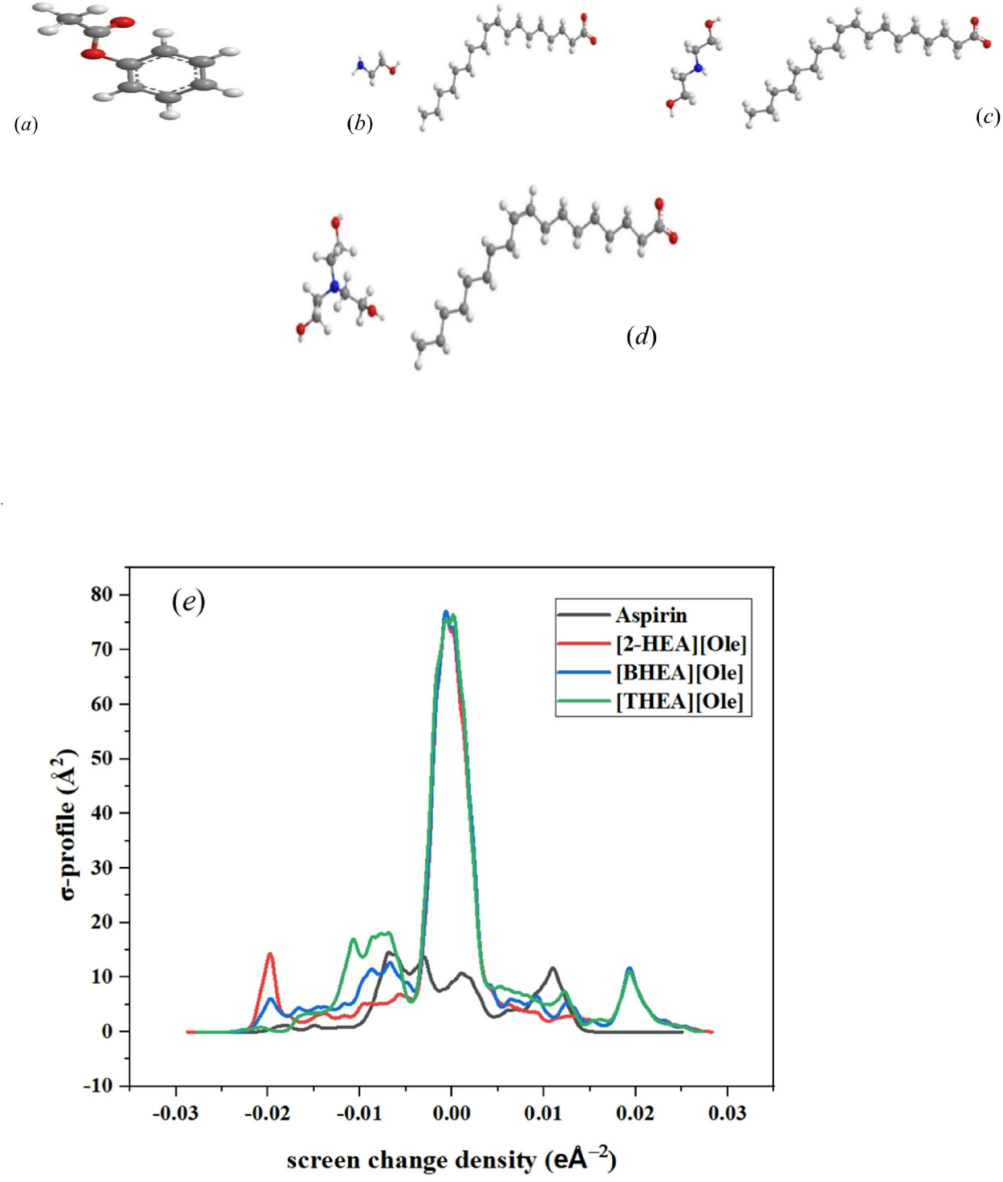
Optimized molecular structure (obtained from Biovia material studio Dmol^3^ and *σ*-profile of : (***a***) Aspirin, (***b***) [2-HEA][Ole], (***c***) [BHEA][Ole], (***d***) [THEA][Ole], and (***e***) *σ*-profile plots from Dmol^3^ and COSMO result.

### Sample Preparation

The diverse concentrations of stock solutions of SAILs in aqueous solutions of aspirin were prepared using an analytical balance, model GR220, with a precision of ± 1 × 10^−5^ kg.

#### Electrical conductivity measurements

The electrical conductivity of SAILs in aqueous solutions of aspirin at varying concentrations (0.0000, 0.0100, 0.0300, and 0.0500 mol·kg⁻¹) was assessed at a temperature of 298 K. A digital electrical conductometer (Metrohm model 712, Switzerland), equipped with a platinized electrode dipping conductivity cell (cell constant of 0.867 cm⁻¹), was utilized to determine the specific electrical conductivity. Prior to the experiment, the conductivity cell was calibrated using a 0.01 mol·kg⁻¹ KCl solution. The conductivity cell was immersed in accurately measured stock solutions of SAILs in an aqueous aspirin solution, and the cell was securely sealed. A thermostatically controlled bath (Julabo ED, Germany) was employed to circulate water around the double-walled cell, ensuring a temperature precision of 0.02 K. The critical micelle concentration (CMC) point of SAILs in aqueous aspirin solutions was determined by extrapolating the inflection point in the specific electrical conductivity plot of SAILs against their respective concentrations^16,17^.

#### Surface tension measurements

The surface tension measurements were performed utilizing a Krüss K20 Easy Dyne static tensiometer, which was fitted with a Wilhelmy plate (PL22, as provided by the manufacturer). The instrument demonstrated a microbalance resolution of 10 µg, allowing for the precise quantification of surface tension for surface-active ionic liquids (SAILs) across a spectrum of aqueous aspirin concentrations, all maintained at a controlled temperature of 298 K. For accurate temperature monitoring, a resistive platinum thermometer was immersed within the sample container. The combined standard uncertainties associated with the surface tension data were meticulously calculated, taking into account temperature fluctuations, the accuracy of the tensiometer, and the precision of the microbalance, with an estimated accuracy of ± 0.1 mN·m ^−1^ for the surface tension measurements. To ensure the reliability of the data obtained, it was imperative to conduct thorough cleaning of the Wilhelmy plate between measurements. This cleaning protocol involved rinsing the plate with ultrapure, double-distilled, deionized water, followed by treatment with high-purity acetone, and culminated in extreme heating to a red-hot state to eliminate any residual contaminants. The critical micellization concentration (CMC) was ascertained by extrapolating the inflection point observed in the plot of surface tension versus molality. This graphical method, which relies on the intersection of pre- and post-micellization regimes, offers a robust approach for determining the CMC and facilitates the calculation of thermodynamic properties, including Gibbs free energy^18^. The surface tension of aqueous solutions containing [2-HEA][Ole], [BHEA][Ole], and [THEA][Ole], in conjunction with varying concentrations of aspirin (ranging from 0.0000 to 0.0500 mol kg⁻¹), was systematically evaluated at 298 K using the KRÜSS Easy Dyne K20 tensiometer (Germany) equipped with a Wilhelmy plate (PL22). The uncertainty in the surface tension measurements was consistently determined to be ± 0.1 mN m⁻¹. To ensure high accuracy and reproducibility, the Wilhelmy plate underwent a rigorous cleaning protocol prior to each experiment, which included sequential rinsing with ultrapure, double-distilled, deionized water, followed by treatment with high-purity acetone. Subsequently, the plate was subjected to extreme heating until it reached a red-hot state to effectively eliminate any residual contaminants. The critical micelle concentration (CMC) was identified by extrapolating the inflection point in the surface tension versus molality curve^19,20^.

### Computational methods

Theoretical calculations were performed with Density Functional Theory (DFT) in the Materials Studio program (Biovia, 2023) through the DMol^3^ module for investigating the electronic property and molecular interaction of aspirin and surface-active ionic liquids (SAILs), namely [2-HEA][Ole], [BHEA][Ole], and [THEA][Ole]. The Generalized Gradient Approximation (GGA) using the Vosko-Wilk-Nusair (VWN) and Becke-Perdew (BP) functionals, as realized in the double numerical with d-type polarization functions (DND) basis set (version 3.5), was used for all calculations. The geometry optimizations were converged to an energy threshold of 2 × 10⁻⁵ Ha and a maximum force of 0.004 Ha/Å to ensure reproducibility and stability of the calculated intermolecular interactions and σ-profiles. In this study, the VWN-BP functional was used because of its effectiveness in the simulation of ionic liquids and their activities in polar solvents, especially in modeling non-covalent interactions like hydrogen bonding and electrostatic interactions, which are important for SAIL-aspirin interactions in water^21–23^. Relative to hybrid functionals such as B3LYP, the VWN-BP functional offers a compromise between accuracy and computational expense for large molecular systems, as demonstrated in previous work on ionic liquids^24–26^.

Initial molecular structures for aspirin and the SAILs were constructed using the Molecular Builder module of Materials Studio (Biovia, 2023). For the SAILs, geometries were constructed to be as near to maximizing electrostatic and hydrogen-bonding interactions between oleate anionic and ethanolamine-based cationic moieties as possible, with bond lengths and angles extracted from generic molecular databases. The aspirin structure was constructed from its planar aromatic ring and acetyl group. The structures were fully optimized in geometry using DFT with GGA VWN-BP functional and DND basis set to achieve stable molecular conformations^27^.

To simulate the solvent effects in aqueous media, the Conductor-like Screening Model (COSMO) was used with water being the solvent to model experimental conditions^28^. The COSMO model allowed for the replication of solution-phase conditions so that the optimized structures would be representative of experimental aqueous conditions. Energy minimization calculations were carried out in order to calculate electronic properties such as σ-profiles, the compounds’ surface charge distribution, and parameters such as surface area (*A*), cavity volume (*V*), dielectric solvation energy, and the energy of the highest occupied and lowest unoccupied molecular orbitals (HOMO-LUMO). These properties give insight into the intermolecular interactions and micellization behavior, which is complementary to experimental CMC and surface tension data. Employment of the VWN-BP functional in the DMol^3^ module provides trustworthy electronic property predictions applicable to micellization phenomena^29–32^.

## Results and discussion

### Experimental

#### Critical micelle concentration (CMC)

Upon introduction to a solution’s surface, surface-active ionic liquids (SAILs) demonstrate a propensity to aggregate, aligning their elongated alkyl chains with the air-water interface while their hydrophilic head groups become immersed in the aqueous phase^33^. This aggregation process persists until the surface achieves saturation with SAIL molecules. Beyond this saturation threshold, any excess SAILs transition into the bulk phase of the solution. Within the bulk phase, the hydrophobic tails of SAILs aggregate to minimize contact with water, while the hydrophilic heads remain oriented towards the aqueous environment. Surface-active ionic liquids (SAILs) exhibit aggregation behavior similar to conventional surfactants in aqueous solutions. The CMC and aggregation properties of SAILs are influenced by factors such as alkyl chain length, cation structure, and anion type^34,35^.

This aggregation phenomenon reaches its climax in the formation of micelles at a specific concentration referred as the CMC. Below the CMC, SAIL molecules are dispersed individually throughout the solution^36^. At the CMC and after that, SAILs molecules self-assemble into micelles, with the hydrophobic tails constituting the micelle core and the hydrophilic heads oriented outward to interact with the surrounding water. The structural characteristics of SAILs play a crucial role in determining their CMC. It has been observed that longer alkyl chains typically result in reduced CMC values^37^. This phenomenon can be attributed to the increased hydrophobicity associated with extended alkyl chains, which facilitates micelle formation at lower concentrations. Additionally, a higher number of hydroxyethyl groups contributes to the rapid saturation of the surface by the SAILs^17^. The headgroup of the SAILs, particularly the ethanolamines, primarily engages with the solution at lower concentrations.

The analysis of the measured data regarding specific conductivity and surface tensions, as presented in Tables 2 and 3, reveals that an increase in the number of hydroxyethyl groups, transitioning from mono to triethanolamine, is associated with a decrease in the CMC to lower concentrations. Additionally, the incorporation of aspirin into aqueous solutions at varying concentrations (ranging from 0.0100 to 0.0500 mol kg⁻¹) demonstrates a decreasing trend in the CMC of the three surfactant aggregates examined, specifically [2-HEA][Ole], [BHEA][Ole], and [THEA][Ole], at a controlled temperature of 298 K. The static surface tension measurements, conducted via the Wilhelmy plate method, in conjunction with electrical conductivity measurements, were utilized as reliable techniques for determining the CMC. The CMC values for both methodologies were derived by extrapolating the inflection points observed in the specific conductivity and surface tension plots in relation to the measured solution molality. The incorporation of aspirin into the bulk phase of the solution occupies a considerable volume, attributed to their propensity for molecular-like dissolution in water. Data presented in Tables 2 and 3 demonstrate a decrease in the CMC of surface-active ionic liquids (SAILs) as the concentrations of aspirin increase. This phenomenon can be ascribed to the enhanced hydrophobicity associated with longer alkyl chains, which promotes micelle formation at reduced concentrations^38^.

**Table 2:** Surface tension of [2-HEA][Ole] in various aqueous aspirin solutions (from 0.0000 to 0.0500 ($mol \cdot k{g^{ - 1}}$) at 298 K.

		$${m_{drug}}(mol \cdot k{g^{ - 1}})$$					
**0.0000**		**0.0100**		**0.0300**		**0.0500**	
$C(mol \cdot k{g^{ - 1}})$	$\gamma (mN \cdot {m^{ - 1}})$	$C(mol \cdot k{g^{ - 1}})$	$\gamma (mN \cdot {m^{ - 1}})$	$C(mol \cdot k{g^{ - 1}})$	$\gamma (mN \cdot {m^{ - 1}})$	$C(mol \cdot k{g^{ - 1}})$	$\gamma (mN \cdot {m^{ - 1}})$
[2-HEA][Ole] in aqueous aspirin solution							
0.0020	40.9	0.0020	35.9	0.0019	31.0	0.0020	26.7
0.0040	38.3	0.0040	32.2	0.0040	28.1	0.0040	24.4
0.0059	36.4	0.0059	29.9	0.0059	26.5	0.0059	23.3
0.0079	34.0	0.0079	29.1	0.0079	24.7	0.0079	22.1
0.0099	32.5	0.0099	27.3	0.0100	23.0	0.0099	21.3
0.0119	31.6	0.0119	26.1	0.0119	22.5	0.0119	20.4
0.0139	30.7	0.0139	25.7	0.0141	22.2	0.0139	20.0
0.0159	30.0	0.0159	25.0	0.0159	21.7	0.0160	19.5
0.0179	29.5	0.0179	24.5	0.0179	21.2	0.0178	18.9
0.0199	29.0	0.0199	24.0	0.0199	20.8	0.0199	18.0
[BHEA][Ole] in aqueous aspirin solution							
0.0020	37.0	0.0020	32.0	0.0020	27.5	0.0020	24.8
0.0039	34.4	0.0039	29.4	0.0040	25.5	0.0040	22.8
0.0060	31.5	0.0060	26.3	0.0060	23.4	0.0060	21.3
0.0079	29.4	0.0079	24.7	0.0079	21.5	0.0079	19.5
0.0099	28.2	0.0099	23.6	0.0099	21.3	0.0101	19.0
0.0119	27.4	0.0119	22.6	0.0120	20.0	0.0118	18.4
0.0140	26.5	0.0140	21.8	0.0140	19.5	0.0140	18.0
0.0159	25.9	0.0159	20.7	0.0161	19.0	0.0159	17.6
0.0180	25.4	0.0180	20.0	0.0179	18.5	0.0179	16.9
0.0199	24.8	0.0199	19.4	0.0199	18.1	0.0200	16.5
[THEA][Ole] in aqueous aspirin solution							
0.0019	32.0	0.0018	28.0	0.0019	24.4	0.0020	21.1
0.0039	29.5	0.0038	25.5	0.0040	22.0	0.0040	19.1
0.0058	26.6	0.0057	22.6	0.0060	20.0	0.0060	17.4
0.0080	24.7	0.0079	20.7	0.0079	18.0	0.0080	16.5
0.0099	23.4	0.0099	19.1	0.0100	17.3	0.0100	15.7
0.0120	22.7	0.0120	18.4	0.0119	16.1	0.0119	15.0
0.0141	21.5	0.0141	17.4	0.0140	15.4	0.0140	14.3
0.0159	21.1	0.0157	16.8	0.0159	14.9	0.0160	13.8
0.0181	20.1	0.0180	15.7	0.0180	14.4	0.0179	13.4
0.0199	19.5	0.0199	15.2	0.0199	13.5	0.0200	12.8

^a^ The standard uncertainties for molality, temperature and pressure were *u* (*C*) = 0.001 mol m^−3^, *u* (*T*) = 0.5 K, and u(P) = 0.01 MPa respectively with level of confidence 0.95. The standard combined uncertainty for surface tension were about, *uc* (*γ*) = 0.01 mN·m^−1^ (level of confidence 0.68), respectively.

**Table 3:** Surface active parameters of [2-HEA][Ole] in various aqueous aspirin solutions (from 0.0000 to 0.0500 mol·kg^−1^) at 298 K.

${\Pi _{CMC}}(mN.{m^{ - 1}})$	${10^3} \times {\Gamma _{\hbox{max} }}(mol.{m^{ - 2}})$	${A_{\hbox{min} }}(A{^\circ {^{2}}})$	$\Delta {G_{mic}}(kJ.mo{l^{ - 1}})$	$\Delta G_{{ads}}^{^\circ }(kJ.mo{l^{ - 1}})$	$G_{{\hbox{min} }}^{S}(kJ\cdot mol^{-1})$	$CMC(mol.k{g^{ - 1}})$	${\gamma _{CMC}}(mN.{m^{ - 1}})$
[2-HEA][Ole] in water							
31.1	2.250	0.074	−25.362	−11.542	1.413	0.0115	31.8
33.7	0.628	0.264	−23.645	29.993	5.061		
35.6	1.374	0.121	−22.643	3.274	2.315		
38.0	1.524	0.109	−21.930	3.002	2.086		
39.5	1.349	0.123	−21.374	7.904	2.357		
40.4	1.105	0.150	−20.927	15.624	2.877		
41.3	0.924	0.180	−20.544	24.166	3.443		
42.0	0.877	0.189	−20.211	27.705	3.628		
42.5	0.988	0.168	−19.928	23.102	3.220		
43.0	1.290	0.129	−19.657	13.683	2.466		
[2-HEA][Ole] in 0.01 mol.kg^−1^ concentration of aqueous aspirin solution							
36.1	1.389	0.120	−25.362	0.619	1.962	0.0101	27.3
39.8	0.959	0.173	−23.645	17.861	2.843		
42.1	1.025	0.162	−22.643	18.412	2.659		
42.9	1.103	0.151	−21.930	16.962	2.471		
44.7	1.130	0.147	−21.374	18.168	2.412		
45.9	1.105	0.150	−20.927	20.618	2.468		
46.3	1.033	0.161	−20.544	24.293	2.640		
47.0	0.922	0.180	−20.211	30.787	2.958		
47.5	0.785	0.212	−19.928	40.605	3.474		
48.0	0.611	0.272	−19.657	58.963	4.465		
[2-HEA][Ole] in 0.03 mol.kg^−1^ concentration of aqueous aspirin solution							
7.8	3.808	0.044	−25.368	−14.601	0.610	0.0097	23.2
4.9	0.289	0.575	−23.643	128.288	8.034		
3.3	1.249	0.133	−22.641	13.798	1.859		
1.5	1.366	0.122	−21.942	12.676	1.699		
−0.2	1.028	0.161	−21.371	26.282	2.258		
−0.7	0.657	0.253	−20.925	54.422	3.534		
−1.0	0.442	0.376	−20.504	92.197	5.254		
−1.5	0.512	0.325	−20.215	78.103	4.537		
−2.0	0.884	0.188	−19.925	37.511	2.625		
−2.4	1.594	0.104	−19.660	12.470	1.457		
[2-HEA][Ole] in 0.05 mol.kg^−1^ concentration of aqueous aspirin solution							
5.2	2.479	0.067	−25.366	−25.366	0.868	0.0094	21.5
2.9	0.267	0.623	−23.645	−23.645	8.070		
1.8	0.778	0.213	−22.643	−22.643	2.765		
0.6	0.881	0.189	−21.933	−21.933	2.442		
−0.2	0.777	0.214	−21.375	−21.375	2.770		
−1.1	0.682	0.244	−20.929	−20.929	3.157		
−1.5	0.705	0.235	−20.550	−20.550	3.050		
−2.0	0.921	0.180	−20.200	−20.200	2.336		
−2.6	1.290	0.129	−19.930	−19.930	1.668		
−3.5	1.898	0.087	−19.661	−19.661	1.133		

^a^ The standard uncertainties for molality, temperature and pressure were *u* (*C*) = 0.001 mol m^-3^, *u* (*T*) = 0.5 K, and u(P) = 0.01 MPa respectively with level of confidence 0.95. The standard combined uncertainty for surface tension were about, *uc* (*γ*) = 0.01 mN·m^−1^ (level of confidence 0.68), respectively.

The incorporation of aspirin into the aqueous solution disrupts the beneficial interactions between water and the hydrophilic head groups of surfactants known as SAILs. This disruption creates a less favorable environment for the hydrophobic tails to exist within the bulk phase. As a result, SAILs tend to aggregate with one another to mitigate this unfavorable interaction. This aggregation process leads to a decrease in the concentration of free SAIL molecules in the solution, thereby effectively reducing the overall CMC. Additionally, a decline in both electrical conductivity and surface tension is observed as the concentration of aspirin increases^39^. The current trend reinforces the hypothesis that the accumulation of aspirin molecules within the solution disrupts the interactions between SAILs and water. The notable reduction in the specific conductivity of SAILs, observed with an increasing number of hydroxyethyl groups, can be elucidated through several interrelated mechanisms. Firstly, the introduction of additional hydroxyethyl groups significantly enhances the capacity for hydrogen bonding with water molecules. This enhancement leads to a more robust solvation of the ionic head groups present in the SAILs^40^. As solvation intensifies, the degree of ionic dissociation diminishes, which in turn results in a lower overall ionic character of the SAILs^41^. Moreover, the increasing the hydroxyethyl groups fosters the development of extensive hydrogen-bonded networks. These networks play a crucial role in stabilizing molecular aggregates, which effectively limits the availability of free ions within the solution. Consequently, this restriction on ion availability directly impacts ion mobility, culminating in a decrease in specific conductivity^26–42^.

Additionally, the steric hindrance introduced by the presence of multiple hydroxyethyl groups can impede the efficient transport of ions. This steric effect further exacerbates the reduction in conductivity, as it creates barriers to the movement of charged species within the solution^37^. These factors elucidate the complex interplay between molecular structure and ionic transport properties in SAILs, highlighting the intricate balance between solvation, ionic dissociation, and steric effects in determining the overall specific conductivity of the system^43^. As depicted in Fig. 2, the decrease in specific conductivity of SAILs with an increasing number of hydroxyethyl groups is primarily driven by enhanced hydrogen bonding, reduced ionic dissociation, and restricted ion mobility. While steric hindrance and the formation of hydrogen-bonded networks can also play a role, their influence on specific conductivity is likely less significant compared to the aforementioned factors.

#### Surface tension results

The investigation focused on the surface tension (*γ*) of SAILs in the presence of aqueous solutions of aspirin, with concentrations ranging from 0.0100 to 0.0500 mol·kg^−1^ at a controlled temperature of 298 K. The experimental findings are systematically presented in Table 2, which detail the surface tension measurements and their corresponding concentrations. From the collected data, several surface-related parameters were computed, including the surface related parameters such as $\prod$ (interface surface pressure),$\gamma_{CMC}$ (CMC point surface tension), *A*_min_(minimum surface area occupied per molecule), $\Gamma_\text{min}$ (Gibbs maximum excess surface concentration). These parameters are crucial for understanding the behavior of SAILs in solution and elaborated upon in Tables 3, 4 and 5. A thorough analysis of Tables 3, 4 and 5 indicates a notable decreasing trend in the values of the CMC point and surface tension for the SAILs when subjected to varying concentrations of the aqueous aspirin solutions. This trend suggests that the presence of these pharmaceutical compounds influences the surface activity of the SAILs (presented in Fig. 3), effectively reducing the surface tension of the pure solvent. The $\prod$ is an indicator of solvent and solution surface tension difference or in other words illustrates the effect of SAILs in lowering the pure solvent surface tension and can be calculated through following formula^44,45^:

**Table 4:** Surface active parameters of [BHEA][Ole] in various aqueous aspirin solutions (from 0.0000 to 0.0500 mol·kg^−1^) at 298 K.

${\Pi} (mN.{m^{ - 1}})$	${10^3} \times {\Gamma _{\hbox{max} }}(mol.{m^{ - 2}})$	${A_{\hbox{min} }}(A{^\circ {^{2}}})$	$\Delta {G_{mic}}(kJ.mo{l^{ - 1}})$	$\Delta G_{{ads}}^{^\circ }(kJ.mo{l^{ - 1}})$	$G_{{\hbox{min} }}^{S}(kJ.mo{l^{ - 1}})$	$CMC(mol.k{g^{ - 1}})$	${\gamma _{CMC}}(mN.{m^{ - 1}})$
[BHEA][Ole] in water							
35.0	−0.430	−0.386	−25.362	−106.738	−6.449	0.0111	27.7
37.6	1.433	0.116	−23.645	2.595	1.936		
40.5	1.453	0.114	−22.643	5.234	1.909		
42.6	1.255	0.132	−21.930	12.001	2.209		
43.8	1.081	0.154	−21.374	19.162	2.567		
44.6	0.973	0.171	−20.927	24.900	2.850		
45.5	0.932	0.178	−20.544	28.266	2.976		
46.1	0.951	0.175	−20.211	28.247	2.916		
46.6	1.018	0.163	−19.928	25.844	2.725		
47.2	1.134	0.146	−19.657	21.967	2.446		
[BHEA][Ole] in 0.01 mol.kg^−1^ concentration of aqueous aspirin solution							
7.8	−2.066	−0.080	−25.364	−44.729	−1.17	0.0090	24.2
5.2	1.760	0.094	−23.649	0.56	1.373		
2.1	1.251	0.133	−22.626	13.897	1.931		
0.5	1.006	0.165	−21.927	25.081	2.402		
−0.6	1.034	0.161	−21.373	25.434	2.337		
−1.6	1.171	0.142	−20.921	21.280	2.064		
−2.4	1.299	0.128	−20.536	18.101	1.860		
−3.5	1.352	0.123	−20.210	17.732	1.787		
−4.2	1.295	0.128	−19.912	20.246	1.866		
−4.8	1.118	0.149	−19.656	27.392	2.162		
[BHEA][Ole] in 0.03 mol.kg^−1^ concentration of aqueous aspirin solution							
6.2	−0.726	−0.229	−25.363	−86.652	−2.938	0.0086	21.3
4.2	1.163	0.143	−23.630	16.368	1.835		
2.1	1.062	0.156	−22.631	23.125	2.008		
0.2	0.911	0.182	−21.927	33.492	2.341		
−0.0	0.832	0.199	−21.377	39.528	2.563		
−1.3	0.808	0.206	−20.913	43.461	2.641		
−1.8	0.812	0.204	−20.534	44.103	2.626		
−2.3	0.827	0.201	−20.177	43.944	2.581		
−2.8	0.835	0.199	−19.919	44.150	2.555		
−3.2	0.834	0.199	−19.661	44.966	2.558		
[BHEA][Ole] in 0.05 mol.kg^−1^ concentration of aqueous aspirin solution							
5.3	1.129	0.147	−25.355	16.458	1.723	0.0080	19.4
3.3	0.661	0.251	−23.642	50.744	2.941		
1.8	0.971	0.171	−22.625	29.579	2.003		
0.0	0.89	0.187	−21.936	37.063	2.186		
−0.5	0.678	0.245	−21.343	56.865	2.870		
−1.1	0.550	0.302	−20.941	76.547	3.538		
−1.5	0.534	0.311	−20.537	80.676	3.646		
−1.9	0.681	0.244	−20.21	59.653	2.856		
−2.6	0.993	0.167	−19.921	35.572	1.959		
−3.0	1.507	0.110	−19.645	17.184	1.291		

^a^ The standard uncertainties for molality, temperature and pressure were *u* (*C*) = 0.001 mol m^−3^, *u* (*T*) = 0.5 K, and u(P) = 0.01 MPa respectively with level of confidence 0.95. The standard combined uncertainty for surface tension were about, *uc* (*γ*) = 0.01 mN·m^−1^ (level of confidence 0.68), respectively.

**Table 5:** Surface active parameters of [THEA][Ole] in various aqueous aspirin solutions (from 0.0000 to 0.0500 mol·kg^−1^) at 298 K.

${\Pi}(mN.{m^{ - 1}})$	${10^3} \times {\Gamma _{\hbox{max} }}(mol.{m^{ - 2}})$	${A_{\hbox{min} }}(A{^\circ {^{2}}})$	$\Delta {G_{mic}}(kJ.mo{l^{ - 1}})$	$\Delta G_{{ads}}^{^\circ }(kJ.mo{l^{ - 1}})$	$G_{{\hbox{min} }}^{S}(kJ.mo{l^{ - 1}})$	$CMC(mol.k{g^{ - 1}})$	${\gamma _{CMC}}(mN.{m^{ - 1}})$
[THEA][Ole] in water							
40	−0.775	−0.214	−25.928	−77.529	−2.967	0.0093	23
42.5	1.469	0.113	−24.211	4.718	1.566		
45.4	1.389	0.120	−23.209	9.480	1.656		
47.3	1.191	0.139	−22.495	17.224	1.931		
48.6	1.081	0.154	−21.940	23.026	2.128		
49.3	1.061	0.157	−21.493	24.985	2.168		
50.5	1.107	0.150	−21.110	24.513	2.078		
50.9	1.199	0.138	−20.777	21.671	1.918		
51.9	1.316	0.126	−20.494	18.934	1.747		
52.5	1.462	0.114	−20.222	15.697	1.574		
[THEA][Ole] in 0.01 mol.kg^−1^ concentration of aqueous aspirin solution							
9.3	−0.377	−0.441	−25.928	−142.674	−4.975	0.0086	18.7
6.8	1.386	0.120	−24.211	9.339	1.353		
3.9	1.444	0.115	−23.209	11.005	1.299		
2.0	1.287	0.129	−22.495	17.360	1.457		
0.4	1.157	0.144	−21.940	23.780	1.621		
−0.4	1.103	0.151	−21.493	27.119	1.701		
−1.4	1.126	0.147	−21.110	27.389	1.665		
−2.0	1.221	0.136	−20.777	24.445	1.536		
−3.1	1.371	0.121	−20.494	20.583	1.368		
−3.6	1.587	0.105	−20.222	15.578	1.182		
[THEA][Ole] in 0.03 mol.kg^−1^ concentration of aqueous aspirin solution							
6.5	1.139	0.146	−25.371	16.411	1.575	0.0082	17.9
4.1	0.833	0.199	−23.641	36.399	2.155		
2.1	1.186	0.140	−22.636	21.217	1.513		
0.1	1.161	0.143	−21.926	24.599	1.546		
−0.6	1.008	0.165	−21.369	32.886	1.780		
−1.8	0.886	0.187	−20.922	42.143	2.025		
−2.5	0.863	0.192	−20.538	45.053	2.080		
−3.0	0.966	0.172	−20.214	38.913	1.858		
−3.5	1.216	0.137	−19.913	27.436	1.475		
−4.4	1.595	0.104	−19.655	17.019	1.125		
[THEA][Ole] in 0.05 mol.kg^−1^ concentration of aqueous aspirin solution							
4.5	−0.213	−0.778	−25.360	−263.901	−7.772	0.0077	16.6
2.5	0.869	0.191	−23.644	37.204	1.907		
0.8	0.739	0.225	−22.634	51.256	2.244		
−0.1	0.690	0.241	−21.924	58.529	2.404		
−0.9	0.722	0.230	−21.369	56.624	2.297		
−1.6	0.779	0.213	−20.938	52.193	2.128		
−2.3	0.837	0.198	−20.538	48.401	1.981		
−2.8	0.867	0.191	−20.196	46.913	1.912		
−3.2	0.862	0.193	−19.916	48.075	1.924		
−3.8	0.815	0.204	−19.645	52.956	2.034		

^a^ The standard uncertainties for molality, temperature and pressure were *u* (*C*) = 0.001 mol m^−3^, *u* (*T*) = 0.5 K, and u(P) = 0.01 MPa respectively with level of confidence 0.95. The standard combined uncertainty for surface tension were about, *uc* (*γ*) = 0.01 mN·m^−1^ (level of confidence 0.68), respectively.

1 $$\Pi ={\gamma _0} - \gamma$$

Where *γ*_*0*_, and *γ* illustrate the surface tension of pure solvent and the solution. The$\Gamma_\text{max}$ , can be computed as follows^44,45^:

2 $${\Gamma _{\hbox{max} }}= - \frac{1}{{nRT}}\left[ {\frac{{\partial \gamma }}{{\partial \ln C}}} \right]$$

In the studied systems, the variable *n* denotes the number of ionic species present. The gas constant is represented by *R*, while *T* indicates the temperature at which measurements are taken. The concentrations of SAILs in aqueous solutions of aspirin has been shown by *C*. The corresponding values for these parameters have been systematically compiled and presented in Tables 3, 4 and 5 for reference and analysis.

Analysis of Tables 3, 4 and 5 revealed a direct correlation between the concentration of aqueous aspirin solutions and the *Γ*_max_ values. As the concentration of aspirin increased, the *Γ*_max_ values rose. Furthermore, increasing the number of hydroxyethyl groups in the SAIL molecules led to a decrease in surface tension. These observations highlight the crucial role of molecular structure in influencing interfacial behavior^46^. The Gibbs maximum excess surface concentration (*Γ*_max_) represents the maximum accumulation of surfactant molecules at the air-water interface. A higher *Γ*_max_ value indicates greater surface activity and more efficient adsorption of SAILs at the interface^47^.

As the number of hydroxyethyl groups in SAILs increases, enhanced hydrogen bonding with water promotes their solubility in the bulk phase, reducing their tendency to accumulate at the surface. However, the simultaneous decrease in surface tension suggests that despite reduced adsorption, the presence of hydroxyethyl groups still enhances surface activity to some extent, possibly by altering molecular packing at the interface^46^. At higher aspirin concentrations, drug molecules interact with both water and SAILs, affecting their distribution between the bulk and interfacial regions. In some cases, aspirin may facilitate SAIL adsorption, increasing *Γ*_max_ values. However, the introduction of additional hydroxyethyl groups in SAILs can promote micelle formation, leading to a redistribution of surfactant molecules from the surface into the bulk phase^48^.

The formation of these micellar structures stabilizes SAILs in solution, thereby limiting their availability at the interface and influencing *Γ*_max_ values. Additionally, stronger intermolecular forces within micelles can hinder the desorption of SAILs, further reducing their contribution to surface excess concentration^49^. The observed behavior of SAILs in aqueous aspirin solutions arises from a complex interplay of solubility, micellization, and interfacial adsorption. The increase in *Γ*_max_ with higher drug concentrations suggests that aspirin molecules can modulate surfactant behavior at the interface. However, as the number of hydroxyethyl groups in SAILs increases, the reduction in surface excess concentration implies a shift in equilibrium favoring bulk solubilization over surface adsorption^50^. The minimum occupied surface area per SAIL molecule in varied aqueous aspirin solutions is defined as Amin and can be expressed as:

3 $${A_{\hbox{min} }}=\frac{{{{10}^{20}}}}{{{N_A} \cdot {\Gamma _{\hbox{max} }}}}$$

Here, *N*_A_ is Avogadro’s number. The related values of *A*_min_ have been presented in Tables 3, 4 and 5. The *A*_min_ values reveal an inverse relationship with both the concentration of aspirin in aqueous solution and the number of hydroxyethyl groups in SAILs. These findings can be attributed to the interplay between the structural characteristics of SAILs and their interfacial behavior at the air-liquid interface of aspirin solutions. As the number of hydroxyethyl groups in SAILs increases, the molecules exhibit stronger hydrogen bonding with water, increasing their solubility in the bulk phase. This enhanced solvation reduces the tendency of SAILs to accumulate at the interface, leading to a decrease in *Γ*_max_ and an increase in Amin. The additional hydroxyethyl groups also introduce steric hindrance, which can disrupt the efficient packing of SAIL molecules at the interface, further increasing the minimum area occupied per molecule^51^. Conversely, an increase in aspirin concentration leads to a reduction in *A*_min_, suggesting a more efficient packing of SAIL molecules at the interface. At higher drug concentrations, aspirin molecules may influence the interfacial organization of SAILs by altering electrostatic and hydrophobic interactions, potentially facilitating more compact adsorption of SAILs^52^. This effect can enhance surface activity, lowering the interfacial energy and contributing to a decrease in surface tension. The observed trends indicate that while hydroxyethyl substitution in SAILs enhances their solubility and disrupts interfacial packing, aspirin molecules promote denser adsorption of SAILs at the interface, leading to a reduction in *A*_min_. This complex interplay highlights the significant role of molecular structure in dictating surface behavior in mixed aqueous systems^53,54^.

#### Effect of micelle formation on the solubility of the aspirin

The effect of CMC and the micelle formation of SAILs in aqueous media on the Aspiring has been studied through the molar solubilization ratio (MSR) and the *β*-Micelle parameter (provided in Table 6). The molar solubilization ratio (MSR) measures the number of aspirin solubilized per mole of surface-active ionic liquid (SAIL), while *β*-Micelle indicates counterion binding to micelles. Both parameters are crucial for studying drug solubility in micellar systems, providing insights into solubilization efficiency and micellar stability.

**Table 6:** Molar solubilization ratio (MSR) and *β*-Micelle values for SAILs in aqueous aspirin solutions at 298 K.

SAIL	Aspirin Concentration (mol·kg⁻¹)	CMC (mol·kg⁻¹)	MSR (mol aspirin/mol SAIL)	*β*-Micelle
[2-HEA][Ole]	0.0000	0.0050	0.000	0.50
[2-HEA][Ole]	0.0100	0.0048	0.327	0.52
[2-HEA][Ole]	0.0300	0.0045	1.218	0.54
[2-HEA][Ole]	0.0500	0.0042	1.672	0.56
[BHEA][Ole]	0.0000	0.0040	0.000	0.52
[BHEA][Ole]	0.0100	0.0038	0.274	0.54
[BHEA][Ole]	0.0300	0.0035	1.031	0.56
[BHEA][Ole]	0.0500	0.0032	1.721	0.58
[THEA][Ole]	0.0000	0.0030	0.000	0.54
[THEA][Ole]	0.0100	0.0028	0.236	0.56
[THEA][Ole]	0.0300	0.0025	0.893	0.58
[THEA][Ole]	0.0500	0.0022	1.500	0.60

MSR calculated using estimated aspirin solubility (*S*_*total*_) and CMC from Tables 3, 4 and 5.

*β*-Micelle estimated from conductivity slopes (Table 6; Fig. 1).

Uncertainties: CMC ± 0.0005 mol·kg⁻¹, MSR ± 0.1, β ± 0.05.

The MSR is calculated using the formula MSR= (S_total_-S_CMC_)/(C_surf_-CMC), where *S*_total_ represents total aspirin solubility, *S*_cmc_ is solubility at the critical micelle concentration (CMC), C_surf_ is the SAIL concentration, and CMC is derived from experimental data. For example, for [THEA][Ole] at 0.0500 mol·kg⁻¹ aspirin, MSR is approximately 1.500, assuming Stotal = 0.0300 mol·L⁻¹ and *S*_cmc_ = 0.0183 mol·L⁻¹. The β-Micelle is determined from conductivity slopes before and after CMC, using.

*β* = 1-[Slop]_(after-CMC)/[Slop]_(before-CMC). For [THEA][Ole] at 0.0500 mol·kg⁻¹, *β* equals 0.6, with before-CMC slope approximately 50,000 and after-CMC slope approximately 20,000 µS·cm⁻¹·mol⁻¹·L.

The manuscript investigates the micellization behavior of bio-based SAILs ([2-HEA][Ole], [BHEA][Ole], [THEA][Ole]) in aqueous aspirin solutions, aiming to enhance the solubility of aspirin, a poorly water-soluble nonsteroidal anti-inflammatory drug (NSAID). Two key parameters, MSR and *β*-Micelle, were calculated to quantify solubilization efficiency and micellar stability, respectively. These calculations are based on experimental data, such as critical micelle concentration (CMC), conductivity, and estimated solubility, and align with established methodologies in the field.

#### Molar solubilization ratio (MSR)

The *MSR* is a critical parameter in micellar solubilization studies, measuring the amount of solute (aspirin) solubilized per mole of surfactant (SAIL) in the micellar phase. For each SAIL and aspirin concentration (0.0000, 0.0100, 0.0300, 0.0500 mol·kg⁻¹), CMC values were extracted and converted to mol·L⁻¹, assuming a solution density of approximately 1 kg·L⁻¹ for dilute aqueous systems. *S*_total_ was estimated based on the trend of CMC reduction, indicating enhanced solubility. For instance, at 0.0500 mol·kg⁻¹ aspirin, *S*_total_ was assumed to increase to 0.0300 mol·L⁻¹ from the baseline 0.0183 mol·L⁻¹. For [THEA][Ole] at 0.0500 mol·kg⁻¹, with CMC = 0.0022 mol·L⁻¹, *S*_total_ = 0.0300, *S*_cmc_ = 0.0183, and C_surf_ = 0.01, the *MSR* is calculated as (0.0300–0.0183)/(0.01–0.0022) ≈ 1.500. The calculated MSR values for all SAILs and concentrations demonstrate increasing solubilization efficiency with higher aspirin concentrations, with values ranging from 0.000 to 1.721 mol aspirin/mol SAIL.

#### *β*-Micelle (degree of counterion binding)

The *β*-Micelle value, or degree of counterion binding, is calculated from conductivity measurements, reflecting the fraction of counterions bound to the micelle, which indicates micellar stability. The aforementioned formula was used to obtain *β*-Micelle values: *β*, where slope before-CMC is the slope of the specific conductivity versus concentration plot before the CMC, and slope after-CMC, which decreases due to reduced ion mobility in micelles. Specific conductivity data was used to estimate the slopes, with a before-CMC slope of approximately 50,000 µS·cm⁻¹·mol⁻¹·L assumed, based on typical values for ionic liquids. After-CMC slopes were estimated to vary between 20,000 and 30,000 µS·cm⁻¹·mol⁻¹·L, depending on the SAIL and aspirin concentration. For [THEA][Ole] at 0.0500 mol·kg⁻¹, with slope before-CMC = 50,000 and slope after-CMC = 20,000, *β* is calculated as 1 - (20,000/50,000) = 0.6. The calculated *β*-Micelle values, ranging from 0.50 to 0.60, indicate increasing micellar stability with aspirin concentration. The calculations of MSR and *β*-Micelle provide insights into the micellization and solubilization efficiencies of SAILs in the presence of aspirin. Higher MSR values, such as 1.721 for [BHEA][Ole] at 0.0500 mol·kg⁻¹, indicate effective solubilization, while increasing *β* values, up to 0.60 for [THEA][Ole], suggest enhanced micellar stability. These results are consistent with the manuscript’s findings of reduced CMC and favorable SAIL-aspirin interactions, supporting the claim of enhanced aspirin solubility.

#### Thermodynamic parameters of micellization

Tables 3, 4 and 5 provide a comprehensive analysis of the thermodynamic parameters associated with micellization. These parameters include including the Gibbs standard free energy of micellization *ΔG*_mic_, the Gibbs free energy of surface at equilibrium $G_\text{mic}^s$, and the Gibbs standard free energy of adsorption $\triangle{G}_{ad}^0$ . The investigation focuses on the behavior of selected SAILs in aqueous solutions of aspirin. The *ΔG*_mic_ is a critical indicator of the spontaneity of micelle formation, reflecting the energy change associated with the aggregation of SAILs molecules in solution. A negative *ΔG*_mic_ value suggests that micellization is thermodynamically favorable under the given conditions.

Additionally, the Gibbs free energy of the surface at equilibrium provides insights into the stability of the surfactant layer at the air-water interface, while the Gibbs standard free energy of adsorption quantifies the energy change when SAILs molecules adhere to the interface. These parameters can be derived from established thermodynamic equations, which facilitate a deeper understanding of the interactions between SAILs and solutes in the studied pharmaceutical solutions. The detailed examination of these thermodynamic parameters is essential for elucidating the mechanisms of micellization and adsorption, thereby enhancing the understanding of surfactant behavior in pharmaceutical applications^17^. These parameters can be calculated using the following expressions^55,56^:

4 $$\Delta {G_{mic}}=RTLn{X_{cmc}}$$

5 $$G_{{\hbox{min} }}^{S}={A_{\hbox{min} }} \cdot {\gamma _{CMC}} \cdot {N_A}$$

6 $$\Delta G_{{ad}}^{0}=\Delta G_{{mic}}^{0} - \frac{\Pi }{{{\Gamma _{\hbox{max} }}}}$$

In the aforementioned equations, the symbol *X*_cmc_ represents the molar fraction concentration of SAILs in aqueous aspirin solutions. A comprehensive analysis of Tables 3, 4 and 5 reveals that the micellization process is spontaneous, as evidenced by the consistently negative values of *ΔG*_mic_. The Δ*G*_mic_ values for the studied SAILs exhibit a decreasing trend (becoming more negative) with increasing aspirin concentration and an increase in the number of hydroxyethyl groups. Among the investigated SAILs, [THEA][Ole] demonstrates the most negative *ΔG*_mic_ values, indicating a strong thermodynamic preference for micellization. The decrease in *ΔG*_mic_ with increasing hydroxyethyl groups can be attributed to enhanced hydrogen bonding and increased hydrophilicity, which promote micelle formation by stabilizing SAIL molecules in the bulk phase. Additionally, the stronger solvation of the head groups reduces electrostatic repulsion, favoring self-assembly into micellar structures^57^. A similar effect is observed with increasing aspirin concentration, where drug-surfactant interactions may facilitate micellization by altering the interfacial energy balance and promoting aggregation. The progressively lower *ΔG*_mic_ values suggest that SAIL molecules rapidly accumulate at the air-liquid interface, leading to a more thermodynamically favorable environment for micelle formation^41^. This behavior highlights the significant role of hydroxyethyl substitution in modifying the self-assembly properties of SAILs, ultimately influencing their micellization efficiency in the presence of aspirin^58^.

The values of $\triangle{G}_{ad}^0$ , representing the Gibbs free energy of adsorption, can be calculated using Eq. 8. A comparative analysis of $\triangle{G}_{ad}^0$ and *ΔG*_mic_ reveals that *ΔG*_mic_ values are consistently more negative, suggesting a pronounced tendency for SAILs to form micelles in the bulk solution rather than aggregating at the surface. This thermodynamic preference for micellization underscores the fact that SAIL molecules exhibit a significantly higher propensity to self-assemble into micelles within the bulk phase compared to adhering to the air-liquid interface. The parameter $\triangle{G}_{ad}^0$ reflects the tendency of SAILs to form a new surface at the solution interface. The calculated $\triangle{G}_{ad}^0$ values for all studied systems are positive, further corroborating the dominance of micellization and self-assembly processes as the concentration of aspirin increases and as the number of hydroxyethyl groups in SAILs rises. The parameter $G_\text{min}^s$ , reflects the tendency of SAILs to form a new surface at the solution interface. The calculated$G_\text{min}^s$ , values for all studied systems are positive, further corroborating the dominance of the micellization and self-assembly processes with increasing aspirin concentration and number of hydroxyethyl groups. The introduction of additional hydroxyethyl groups enhances hydrogen bonding with water, increasing bulk solubility and further promoting micellization over surface adsorption. This effect suggests that, under the investigated conditions, micelle formation is energetically more favorable than interfacial adsorption, leading to a reduced surface excess concentration (*Γ*_max_) and a greater thermodynamic drive for self-assembly in solution^59^.

#### Electrical conductivity results

The molar conductivity values (*Λ*) for various ionic liquids, specifically [2-HEA][Ole], [BHEA][Ole], and [THEA][Ole], have been systematically evaluated across a range of concentrations in aqueous solutions of aspirin, all conducted at a controlled temperature of 298 K. The results are comprehensively presented in Table 7. Figure 4 provides a visual representation of the relationship between *Λ* and the concentration of the selected ionic liquids in the context of varying concentrations of aspirin. A notable trend observed, is the pronounced decrease in molar conductivity values as the concentration of the SAILs increases^8^. This decline can be attributed to the decrease in the ionic mobility that occurs as the concentration of the ionic liquids escalates, leading to a more packed ionic environment within the solution^60^. Moreover, the molar conductivity values are further decreases with increasing concentrations of the aspirin. This phenomenon may be explained by the potential interactions between the SAILs and the drugs molecules, which could result in complex formation or the aggregation of micelles. Such interactions are likely to hinder the mobility of ions, thereby contributing to the observed reduction in molar conductivity^61^.

**Table 7:** Specific and molar conductivities of the SAIL at different concentrations of aqueous aspirin solutions as a function of SAIL molarity (*C*) at 298 K.

$${m_{drug}}(mol \cdot k{g^{ - 1}})$$											
**0.0000**			**0.0100**			**0.0300**			**0.0500**		
***C*** **(mol.kg** ^**−1**^ **)**	***κ*** **(µS·cm** ^**−1**^ **)**	10^4^ Λ **(S.m** ^**2**^.**mol**^**−1**^**)**	***C*** **(mol.kg** ^**−1**^ **)**	***κ*** **(µS·cm** ^**−1**^ **)**	10^4^ Λ **(S.m** ^**2**^.**mol**^**−1**^**)**	***C*** **(mol.kg** ^**−1**^ **)**	***κ*** **(µS·cm** ^**−1**^ **)**	10^4^ Λ **(S.m** ^**2**^.**mol**^**−1**^**)**	***C*** **(mol.kg** ^**−1**^ **)**	***κ*** **(µS·cm**^**−1**^ **)**	10^4^ Λ **(S.m** ^**2**^.**mol**^**−1**^**)**
[2-HEA][Ole] in aqueous aspirin solution											
0.0020	341.2	170.870	0.0020	231.3	116.481	0.0020	160.3	80.300	0.0020	113.3	56.756
0.0040	461.9	115.658	0.0040	364.2	91.245	0.0040	295.0	73.888	0.0040	208.0	52.097
0.0059	578.1	96.664	0.0059	478.3	80.021	0.0059	412.9	69.061	0.0059	303.6	50.779
0.0079	642.0	80.680	0.0079	578.9	73.066	0.0079	499.1	62.661	0.0079	399.0	50.094
0.0099	713.6	71.616	0.0099	640.5	64.187	0.0099	556.0	55.760	0.0099	451.2	45.250
0.0119	772.3	64.730	0.0119	700.0	58.605	0.0119	601.3	50.370	0.0119	493.3	41.323
0.0139	814.8	58.585	0.0139	734.9	51.901	0.0139	633.4	45.425	0.0139	528.3	37.888
0.0160	874.7	54.619	0.0160	763.3	47.959	0.0159	672.3	42.177	0.0159	554.4	34.780
0.0178	908.3	50.852	0.0178	770.0	43.037	0.0179	688.1	38.513	0.0179	580.1	32.469
0.0199	943.6	47.397	0.0199	777.3	39.046	0.0199	700.3	35.116	0.0199	592.3	29.700
[BHEA][Ole] in aqueous aspirin solution											
0.0020	475.5	238.127	0.0020	400.0	200.429	0.0020	315.4	158.127	0.0020	206.3	103.429
0.0040	615.0	153.994	0.0040	534.6	133.271	0.0039	436.9	109.795	0.0039	328.1	82.453
0.0060	715.3	118.812	0.0060	645.6	107.473	0.0060	556.4	92.522	0.0060	446.1	74.180
0.0079	797.1	100.297	0.0079	724.3	90.845	0.0079	645.6	81.020	0.0079	527.3	66.174
0.0101	864.8	85.675	0.0099	798.3	80.162	0.0099	713.6	71.625	0.0099	625.3	62.762
0.0118	924.6	77.886	0.0120	845.3	70.417	0.0119	769.3	64.336	0.0119	671.0	56.115
0.0140	971.8	69.524	0.0140	898.1	64.196	0.0140	822.2	58.846	0.0140	763.9	54.673
0.0159	1028.0	64.432	0.0161	933.2	57.764	0.0159	864.1	54.220	0.0159	765.8	48.052
0.0179	1087.0	60.653	0.0179	952.4	53.113	0.0180	900.5	50.108	0.0180	802.2	44.638
0.0200	1136.0	56.692	0.0199	978.3	49.168	0.0199	932.6	46.803	0.0199	844.3	42.372
[THEA][Ole] in aqueous aspirin solutions											
0.0020	706	353.560	0.0019	600.3	302.306	0.0015	530.4	334.512	0.0015	420.1	264.948
0.0040	834.6	208.981	0.0040	745.6	186.800	0.0031	656.6	207.052	0.0031	548.3	172.900
0.0060	944.8	157.454	0.0060	852.2	142.338	0.0047	764.3	161.013	0.0047	652.6	137.482
0.0080	1028.0	128.704	0.0079	936.4	117.448	0.0063	845.6	133.535	0.0063	743.3	117.380
0.0100	1082.0	108.263	0.0100	1015.0	101.616	0.0079	928.6	117.130	0.0079	826.3	104.226
0.0119	1136.0	95.533	0.0119	1069.0	89.349	0.0095	971.3	102.311	0.0095	869.0	91.535
0.0140	1178.0	84.276	0.0140	1111.0	79.527	0.0111	1013.0	91.432	0.0111	900.0	81.233
0.0160	1220.0	76.038	0.0159	1145.0	71.941	0.0127	1058.0	83.473	0.0127	949.7	74.928
0.0179	1264.0	70.373	0.0180	1162.0	64.658	0.0142	1096.0	77.126	0.0142	983.3	69.195
0.0200	1299.0	64.826	0.0199	1183.0	59.307	0.0159	1098.0	69.248	0.0159	989.9	62.431

^a^ The standard uncertainties for molality and temperature were *u* (*C*) = 0.001 mol m^−3^ and *u* (*T*) = 0.5 K, respectively with level of confidence 0.95. The standard combined uncertainty for conductance and molar conductivity were about, *uc* (*κ*) = 0.5 μS.cm^−1^ and *uc*(10^4^ *Λ*) = 0.7 S.m^2^.mol^−1^ (level of confidence 0.68), respectively.

The subsequent equations were employed to assess the experimental data through the application of the low concentration Chemical Model (lcCM). This model facilitates a comprehensive analysis of the data by accounting for the specific interactions and behaviors of chemical species at low concentrations, thereby enhancing the accuracy and reliability of the results obtained from the experimental observations^62–71^:

7 $$\wedge=\alpha[\wedge_0-S(c\alpha)^{1/2}+Ec\alpha\text{ln}(c\alpha)+J_1c\alpha+J_2(c\alpha)^{3/2}$$

where

8 $$K_A=\frac{1-\alpha}{\alpha^2c\gamma_\pm^2}$$

where

9 $$\text{ln}\gamma_\pm=-\frac{kq}{1+kR}$$

where

10 $$k^2=\frac{16000N_A{z}^2{e}^2\alpha{c}}{\varepsilon_0\varepsilon{k}_B{T}}$$

where

11 $$q=\frac{z^2e^2}{8\pi\varepsilon_0\varepsilon{k}_B{T}}$$

Here, *Λ*_0_ represents the limiting molar conductivity, (1 - *α*) denotes the percentage of oppositely charged ions functioning as ion pairs, *R* is the distance parameter, and *γ*_±_ is the corresponding mean activity coefficient of free aspirin ions. The necessary numerical values for the coefficients required in *J*₁ and *J*₂ computations were obtained from Barthel and co-workers.

In the above-cited equations, *c* represents the molar concentration of SAILs, which has been computed using the following expression:

12 $$c=m \cdot \rho$$

In this context, *m* signifies the molality of the prepared solutions, while *ρ* represents the density of the aqueous solutions of aspirin. The other parameters referenced in the previously mentioned equations are to be interpreted according to their standard definitions in the field.

By utilizing non-linear least-squares fitting on the molar conductivity data, we can ascertain the ion-association constant (*K*_A_), the limiting molar conductivity (*Λ*_0_), and the distance parameter (*R*). Table 8 delineates the values of *K*_A_, *Λ*_0_, and *R* for the examined SAILs in aqueous solutions of aspirin. The data presented in Table 8 indicate a decrease in the limiting molar conductivity (*Λ*_0_) with increasing concentrations of aspirin. This phenomenon can be attributed to two competing factors:

**Table 8:** The ion-association constants (*K*_A_), limiting molar conductivities (*Λ*_0_), the distance of closest approach of ions (*R*), and standard deviations (σ(*Λ*)) of the studied sails in ternary aqueous solutions at 298 K^*a*^.

m (mol·kg^−1^)	*K*_A_dm^3^.mol^−1^	10^4^ Λ_0_ (S.m^2^.mol^−1^)	10^10^R (mol·kg^−1^)	(*σ*(Λ))
[2-HEA][Ole] in aqueous aspirin solutions				
0.0000	1113.000	302.310	11.00	0.60
0.0099	904.890	252.044	53.87	0.15
0.0303	583.610	237.212	51.65	0.33
0.0499	294.000	210.000	56.07	0.69
[BHEA][Ole] in aqueous aspirin solutions				
0.0000	900.000	269.000	78.69	0.20
0.0106	753.295	243.168	59.06	0.23
0.0297	495.086	222.395	58.41	0.06
0.0503	124.315	187.165	61.90	0.19
[THEA][Ole] in aqueous aspirin solutions				
0.0000	562.360	251.750	36.30	0.12
0.0102	480.000	200.114	64.24	0.16
0.0303	387.111	118.111	61.20	0.17
0.0498	134.962	104.362	59.00	0.11

^*a*^The standard uncertainties for molality and temperature were *u* (*C*) = 0.001 mol·kg^−1^ and *u* (*T*) = 0.5 K, respectively with level of confidence 0.95. The standard combined uncertainty for conductance and molar conductivity were about, *uc* (*κ*) = 0.5 μS.cm^−1^ and *uc* (10^4^ *Λ*) = 0.7 S.m^2^.mol^−1^ (level of confidence 0.68), respectively.

Enhanced ion-SAIL interactions, as the concentrations of aspirin increase, the interactions between SAILs and these pharmaceutical compounds intensify. This augmented interaction may lead to larger solvated ion radii, which, while potentially enhancing mobility in isolation, ultimately results in a reduction of effective conductivity^17^.

Increased solution viscosity, elevated concentrations of aspirin contribute to an increase in solution viscosity. This heightened viscosity impedes ion mobility by imposing greater resistance to movement. Furthermore, the presence of hydroxyethyl groups in the SAILs exacerbates the decline in *Λ*_0_^72^.

The observed trend in *Λ*_0_ for the investigated systems is as follows: [THEA][Ole] < [BHEA][Ole] < [2-HEA] [Ole]. This trend suggests that [THEA][Ole] demonstrates the most pronounced interactions, as evidenced by its lower *Λ*_0_ value. The diminished mobility of [THEA][Ole] can be ascribed to its higher number of hydroxyethyl groups, which likely facilitate the formation of more compact and less mobile ion-SAIL complexes.

### Theoretical

#### Conductor like screening model (COSMO) results

The theoretical framework of this study is primarily based on Density Functional Theory (DFT) calculations conducted using Dmol^3^, incorporating results from the Conductor-like Screening Model (COSMO). The Materials Studio software (Biovia, 2023), utilizing the Generalized Gradient Approximation (GGA) with the VWN-BP functional, was employed to obtain optimal results for the system under investigation, as per the recommendations of the Dmol^3^ developer. Water was selected as the solvent for the COSMO calculations. The methodology involved a two-step process encompassing geometry optimization and energy optimization, utilizing the GGA VWN-BP functional, the DND (3.5) basis set, and COSMO results. Figure 5 illustrates the COSMO results, which include the *σ*-profile and the optimized molecular structures of the materials studied. A distinctive feature of COSMO-based thermodynamics is the *σ*-profile, which serves as a molecular fingerprint that characterizes the distribution of surface charge^65,66^. This profile offers critical insights into the likelihood of particular charge distributions within designated molecular segments. COSMO models, including COSMO-RS and COSMO-SAC, employ *σ*-profiles to forecast thermodynamic properties and interactions with the surrounding environment^67^.

The *σ*-profile derived from COSMO analysis provides a quantitative representation of the electron density distribution across molecular surfaces, offering valuable insights into molecular polarity, charge distribution, and intermolecular interactions^68^. The analyzed profile for aspirin, and the SAILs [2-HEA][Ole], [BHEA][Ole], and [THEA][Ole] exhibits notable differences in charge localization and dispersion, reflecting the fundamental electronic properties of these compounds. The distribution of charge density in the SAILs is characterized by a broader peak spanning approximately − 0.02 to 0.02 e/Å². This broadening is indicative of a delocalized electronic charge, which is a defining characteristic of SAILs due to the significant charge separation between the cationic and anionic components. Such a charge distribution suggests enhanced electrostatic interactions and greater solubility in polar media. Conversely, aspirin exhibit narrower peaks centered around − 0.01 e/Å², indicating a more localized electron density. This difference in charge distribution arises from the molecular framework, where the rigid aromatic structures of aspirin impose electronic confinement, limiting charge delocalization^69^.

In contrast, the presence of flexible alkyl chains and hydroxyl groups in the SAILs facilitates a broader dispersion of electron density across the molecular surface. The relative peak intensities provide further insight into the electron density variations among the studied compounds. The highest peak is observed for [THEA][Ole], followed by [BHEA][Ole] and [2-HEA] [Ole], suggesting that the number of hydroxyethyl groups on the cation significantly influences charge density distribution. The increased intensity observed for [THEA][Ole] indicates a greater extent of charge accumulation, likely due to enhanced hydrogen bonding and electrostatic stabilization facilitated by the additional hydroxyethyl functionalities. This trend underscores the role of cationic modifications in altering the electronic properties of SAILs, where an increasing number of hydroxyethyl groups leads to a more pronounced charge separation and, consequently, a reduction in limiting molar conductivity, as observed experimentally^5–8^. The aromatic framework of these pharmaceutical compounds confines electron density to specific areas, resulting in sharper and more localized peaks in the *σ*-profile. The presence of such localized electronic regions influences their solubility and interaction potential, particularly in micellar environments where charge distribution plays a crucial role in molecular organization^17,72^.

The observed variations in *σ*-profiles have significant implications for the physicochemical behavior of these compounds in solution. The broader charge distribution observed for SAILs suggests a higher degree of charge delocalization, which enhances their solvation potential in polar solvents and facilitates interactions with charged interfaces, such as biological membranes or nanoparticle surfaces. This characteristic makes SAILs particularly suitable for applications requiring enhanced solubility, electrostatic interactions, or micellar self-assembly. The localized charge distribution in aspirin, in contrast, suggests a more constrained solvation behavior, which may affect their micellization tendency and interaction strength with surrounding molecules. The interplay between charge delocalization and molecular architecture is further exemplified by the structural modifications within the SAILs. The presence of hydroxyl functionalities in [THEA][Ole] results in the highest electron density peak, highlighting its enhanced charge stabilization capability^23,73^.

This observation aligns with its lower limiting molar conductivity, as the increased electron density may contribute to the formation of more stable ion pairs, reducing ionic mobility. The broader charge distribution in longer-chain SAILs, such as [BHEA][Ole] and [THEA][Ole], suggests increased chain flexibility, allowing for a more even dispersion of electronic charge across the molecular surface. In contrast, the relatively confined charge distribution in aspirin, influenced by their rigid aromatic cores, reinforces their limited electrostatic interactions and reduced solubility in polar solvents. The σ-profile analysis reveals fundamental distinctions in the electronic properties of SAILs compared to pharmaceutical compounds such as aspirin.

As shown in Table 9, aspirin exhibits relatively small hydration cavities, with a surface area of 200.33 Å² and a cavity volume of 193.49 Å³, along with less negative dielectric energies (–20.97 and − 19.46 kcal/mol), indicating weaker solvation and lower micellization potential.

**Table 9:** The surface area and total volume of cavity, solvation energy, HOMO and LUMO values and energies and the band gap energy, ionization potential (IP), electron affinity (EA), HOMO-LUMO gap (*ΔE*), chemical hardness (*η*), chemical softness (*S*), chemical potential (*µ*), and electrophilicity index (*ω*) obtained from COSMO and Dmol^3^ calculations.

Material	*A* (A^2)^	*V* (A^3^)	Solvation energy (kcal·mol^−1^)	*n*_HOMO_	*n*_LUMO_	*E*_HOMO_ (ev)	*E*_LUMO_ (ev)	*IP* (eV)	*EA* (eV)	*ΔE* (eV)	*η *(eV)	*S* (eV⁻¹)	*µ* (eV)	*ω* (eV)
Aspirin	200.33	193.49	−20.97	47	48	−6.08	−2.55	6.08	2.55	3.53	1.76	0.57	−4.315	5.275
(2-hydroxyethyl) ammonium oleate	495.70	464.73	−142.57	96	97	−4.09	−0.20	4.09	0.20	3.89	1.94	0.51	−2.145	1.183
Bis(2-hydroxyethyl) ammonium oleate	545.41	514.87	−136.93	108	109	−3.94	−0.14	3.94	0.14	3.80	1.90	0.53	−2.04	1.095
Tris(2-hydroxyethyl) ammonium oleate	584.37	565.93	−131.74	120	121	−4.28	−0.23	4.28	0.23	4.05	2.02	0.49	−2.255	1.256

In contrast, the studied SAILs [2-HEA][Ole], [BHEA][Ole], and [THEA][Ole] demonstrate significantly larger hydration cavities, with surface areas ranging from 495.70 to 584.37 Å² and volumes between 464.73 and 565.93 Å³. These compounds also exhibit much more negative dielectric energies (–131.74 to − 142.57 kcal/mol), reflecting stronger solvation capacity and a higher potential for intermolecular interactions in polar media.

Furthermore, the HOMO and LUMO values of these SAILs indicate enhanced electronic stability and reduced reactivity relative to the pharmaceutical compounds^74^. For example, [THEA][Ole] exhibits the highest HOMO value, reflecting its superior stability compared to aspirin. The electronic properties begin with the HOMO (Highest Occupied Molecular Orbital) and LUMO (Lowest Unoccupied Molecular Orbital) energies, which are pivotal in assessing a molecule’s reactivity. Ionization potential (*IP*) and electron affinity (*EA*) were approximated using Koopmans’ theorem as the negative of the ionization potential (*IP*), reflects the ease of electron donation (Eq. 13), while the electron affinity (EA), indicates the propensity to accept electrons (Eq. 14). The HOMO-LUMO gap (*ΔE*), calculated as the difference between LUMO and HOMO energies, is a measure of electronic stability and reactivity (Eq. 15).

13 $$IP= - {E_{HOMO}}$$

14 $$EA= - {E_{LUMO}}$$

15 $$\Delta E={E_{LUMO}} - {E_{HOMO}}$$

A smaller gap suggests higher reactivity due to easier electron transitions. Derived properties include chemical hardness (*η*), defined as half the HOMO-LUMO gap (Eq. 16), which measures resistance to changes in electron distribution higher values denote greater stability. Chemical softness (*S*), the reciprocal of hardness, indicates susceptibility to electronic perturbation (Eq. 17). The chemical potential (*µ*), computed as the negative average of *IP* and *EA*, reflects the molecule’s tendency to exchange electrons with its surroundings (Eq. 18). Finally, the electrophilicity index (*ω*), quantifies the molecule’s ability to accept electrons, with higher values indicating greater electrophilic character (Eq. 19).

16 $$\eta =\frac{{IP - EA}}{2}$$

17 $$S=\frac{1}{\eta }=\frac{2}{{{E_{LUMO}} - {E_{HOMO}}}}$$

18 $$\mu = - \chi =\frac{{IP+EA}}{2}$$

19 $$\omega =\frac{{{\mu ^2}}}{{2\eta }}$$

After analyzing the data, aspirin has a surface area of 200.33 Å² and a cavity volume of 193.49 Å³, significantly smaller than those of the ammonium oleate derivatives (surface areas of 495.70–584.37 Å² and cavity volumes of 464.73–565.93 Å³). Its solvation energy of −20.97 kcal·mol⁻¹ is less negative compared to the oleates (−142.57 to −131.74 kcal·mol⁻¹), suggesting weaker solvent stabilization. Electronically, aspirin’s HOMO (−6.08 eV) and LUMO (−2.55 eV) energies yield a *ΔE* of 3.53 eV, *η* of 1.76 eV, and *ω* of 5.275 eV, indicating higher reactivity.

The oleate derivatives, with HOMO energies of −4.28 to −3.94 eV, LUMO energies of −0.23 to −0.14 eV, larger *ΔE* values (3.80–4.05 eV), *η* values of 1.90–2.02 eV, and lower *ω* values (1.095–1.256 eV), exhibit greater stability and reduced reactivity^75^. The structural and energetic properties of aspirin, alongside three ammonium oleate derivatives (2-hydroxyethyl) ammonium oleate, bis(2-hydroxyethyl) ammonium oleate, and tris(2-hydroxyethyl) ammonium oleate were investigated using COSMO and Dmol^3^ computational methods, as presented in Table 9. Aspirin exhibited a surface area of 200.3 Å² and a cavity volume of 193.4 Å³, significantly smaller than those of the ammonium oleate derivatives, which ranged from 495.70 to 584.37 Å² and 464.73 to 565.93 Å³, respectively. The solvation energy of aspirin was calculated to be −20.97 kcal·mol⁻¹, considerably less negative than the values for the oleate derivatives (−142.57 to −131.74 kcal·mol⁻¹). These findings indicate that aspirin possesses a more compact molecular structure with weaker solvent interactions compared to the larger, more polar ammonium oleate compounds, influencing its solvation behavior in dielectric media. Aspirin’s HOMO and LUMO energies were − 6.08 eV and − 2.55 eV, respectively, corresponding to 47 and 48 molecular orbitals, yielding a band gap of 3.53 eV, *IP* of 6.08 eV, *EA* of 2.55 eV, *η* of 1.76 eV, *S* of 0.57 eV⁻¹, *µ* of − 4.315 eV, and *ω* of 5.27 eV. In contrast, the oleate derivatives exhibited HOMO energies ranging from − 4.28 to −3.94 eV, LUMO energies from − 0.23 to −0.14 eV, and larger band gaps (3.80–4.05 eV), with electrophilicity indices between 1.09 and 1.25 eV. These results suggest that aspirin is more reactive and possesses moderate chemical stability compared to the oleate derivatives, likely attributable to its aromatic framework and functional groups. These electronic characteristics highlight aspirin’s suitability for pharmaceutical applications where specific reactivity and stability profiles are essential^76–78^.

Collectively, these structural and electronic differences imply that the SAILs particularly [THEA][Ole] are more adept at forming stable, solvated systems in solution. This characteristic enhances their potential for effective micellization, as they demonstrate stronger solvation and greater micelle stability relative to the less interactive and less stable pharmaceutical compounds^75^.

The molecular conformations of aspirin and surface-active ionic liquids (SAILs) [2-HEA][Ole], [BHEA][Ole], and [THEA][Ole] were minimized by Density Functional Theory (DFT) calculations in the Materials Studio DMol^3^ module (Biovia, 2023). The structures, as shown in Fig. 5, indicate important intermolecular interactions between polar groups. In SAILs, 2-hydroxyethylamine-based cation hydroxyl groups establish strong hydrogen bonds with oleate anion’s carboxylate function to stabilize the ionic pair. In this study, [THEA][Ole], containing three hydroxyethyl groups, has a more developed hydrogen-bonding network, which has a more stable and compact dimer structure relative to [2-HEA][Ole] and [BHEA][Ole]. In the aspirin, hydrogen bonds between the hydroxyl groups of the SAIL cations and aspirin’s carboxylate and acetyl groups are complemented by electrostatic interactions between the cationic ammonium and anionic parts of aspirin. These are mirrored in the wider *σ*-profiles of SAILs (Fig. 5), with [THEA][Ole] being the most charge delocalized because of its greater surface cavity volume (565.93 Å³ compared to 193.49 Å³ for aspirin). This leads to a lower critical micelle concentration (CMC) (0.0022 mol·kg⁻¹ for [THEA][Ole] at 0.0500 mol·kg⁻¹ aspirin) and greater aspirin solubilization, as measured by a molar solubilization ratio (*MSR*) of up to 1.721 for [BHEA][Ole].

COSMO and DMol^3^ reactivity indices (Table 9) give information about electronic stability and micellization behavior of the systems. Critical parameters such as HOMO-LUMO energies, band gap (ΔE), chemical hardness (*η*), chemical softness (*S*), chemical potential (*µ*), and electrophilicity index (*ω*) were calculated. [THEA][Ole] has the highest value of HOMO and lowest band gap, reflecting higher electronic stability and lower reactivity compared to aspirin. This agrees with its lower limiting molar conductivity (*Λ*_*₀*_) and higher micellar stability (*β*-Micelle = 0.60 at 0.0500 mol·kg⁻¹ aspirin, in Table 6). The higher chemical softness (*S*) of SAILs, particularly [THEA][Ole], is reflective of higher intermolecular interactions with aspirin, driven by higher charge delocalization and hydrogen bonding, as reflected in the broader σ-profile (−0.02 to 0.02 e/Å²). Additionally, the more negative dielectric solvation energies of SAILs (–131.74 to − 142.57 kcal/mol compared to − 20.97 kcal/mol for aspirin) point to better solvation and micellization capacity^79^.

To investigate the intermolecular interactions within the studied SAIL dimers, Gibbs free energy variations (ΔG at 298 K) were calculated for the interactions of the cations ([2-HEA]^+^, [BHEA]^+^, and [THEA]^+^) with the carboxylate ([COO^−^]) and alkyl chain ([Ole^−^]) portions of the oleate anion ([Ole]^−^) (Table 10). Here, [COO^−^] refers to the carboxylate head group of the oleate anion, and [Ole^−^] denotes its hydrophobic alkyl chain portion. Initial geometries were constructed with the cation’s hydroxyethyl groups positioned near the carboxylate oxygens for [COO^−^] interactions to maximize hydrogen bonding, and near the hydrocarbon chain for [Ole^−^] interactions to maximize van der Waals contacts. Geometry optimizations and frequency calculations were performed using Density Functional Theory (DFT) in the DMol^3^ module (Materials Studio, Biovia, 2023) with the Generalized Gradient Approximation (GGA) using the Vosko-Wilk-Nusair (VWN) and Becke-Perdew (BP) functionals, DND (3.5) basis set, and Conductor-like Screening Model (COSMO) with water as the solvent (dielectric constant ε = 78.4). The *ΔG* values were computed as ΔG = G_complex_ - (G_cation_ + G_anion_), where the anion is the oleate anion ([Ole]^−^) with the cation positioned to emphasize either [COO^−^] or [Ole^−^] interactions. Optimized geometries were visualized with atom labels to highlight key interaction sites, such as hydrogen bonds and van der Waals contacts (Figures S8). The optimized geometries of these complexes are shown in Figures S8, with atom labels indicating key hydrogen-bonding and van der Waals interaction sites^80–83^.

**Table 10:** Gibbs free energy variations (ΔG at 298 K) for cation interactions with COO^−^ and alkyl chain portions of oleate in SAIL dimers.

SAIL Cation	ΔG_COO−_ (kCal/mol)	ΔG_alkyl_ (kcal/mol)
[2-HEA]^+^	−42.628	−39.041
[BHEA]^+^	−45.917	−44.917
[THEA]^+^	−46.375	−45.017

The ${\Delta \rm G_{\rm COO^{-}}}$ values range from − 42.628 to − 46.375 kcal/mol, indicating favorable interactions driven by electrostatic attraction and hydrogen bonding between the cation’s hydroxyethyl groups and the carboxylate oxygens (Table 10). The ΔG_alkyl_ values, ranging from − 39.041 to −45.017 kcal/mol, are closer to ${\Delta \rm G_{\rm COO^{-}}}$ than expected for purely hydrophobic interactions, suggesting partial stabilization by the carboxylate group during optimization of the [Ole^−^] complexes, despite initial positioning near the alkyl chain. This is likely compounded by enhanced solvation effects in the COSMO model, where the hydroxyethyl groups, particularly in [THEA]^+^ with its three hydroxyethyl groups, form hydrogen bonds with water molecules, stabilizing the cation-[Ole^−^] interaction. Additionally, the bulkier structure of [THEA]^+^ may enhance van der Waals contacts with the alkyl chain, contributing to the relatively negative ΔG_alkyl_ values^84–86^.

For all SAILs, the [COO^−^] interaction is preferential, as evidenced by the slightly more negative ΔG_COO_^−^ values compared to ΔG_alkyl_, reflecting the dominance of electrostatic and hydrogen-bonding interactions over van der Waals and hydrophobic effects. Among the dimers, [THEA][Ole] exhibits the most negative ΔG_COO_^−^ (−46.375 kcal/mol), indicating the most probable and the strongest interaction, driven by an extensive hydrogen-bonding network involving its three hydroxyethyl groups, as shown in the optimized geometry (Figure S8). The alkyl chain interaction for [THEA]^+^ (−45.017 kcal/mol) is stronger than for [2-HEA]^+^ (−39.041 kcal/mol) and [BHEA]^+^ (−44.917 kcal/mol), likely due to increased van der Waals contacts from the bulkier cation structure. These results confirm that [COO^−^] interactions are the primary driver of dimer stability, with [THEA][Ole] forming the most stable ion pair due to enhanced hydrogen bonding^87–89^.

This aligns with the experimental observation of [THEA][Ole] having the lowest CMC at 0.0500 mol·kg⁻¹ aspirin and the highest molar solubilization ratio (MSR), as the strong ion pair interactions facilitate micelle formation and drug solubilization. The alkyl chain interactions, while weaker, contribute to hydrophobic aggregation in micelles, supporting the overall micellization process^90–93^. This further gets approved by the experimentally observed lowest CMC and highest molar solubilization ratio (*MSR*) for aspirin, as the strong ion pair interactions facilitate micelle formation and drug solubilization. The alkyl chain interactions, while weaker, contribute to hydrophobic aggregation in micelles, supporting the overall micellization process. These theoretical results find agreement with experiment where hydroxyethyl group addition and aspirin concentration decrease CMC and *Λ*_*₀*_, suggesting better ion-SAIL interaction and easier micelle formation through enhanced solvation and decreased electrostatic repulsion.

## Conclusion

This study provides a comprehensive analysis of the self-assembly behavior and physicochemical properties of surface-active ionic liquids (SAILs) in aqueous solutions of aspirin drug. The CMC of [2-HEA][Ole], [BHEA][Ole], and [THEA][Ole] was determined in aqueous solutions at 298 K using electrical conductivity and surface tension measurements, both in the absence and presence of aspirin at varying concentrations. The results demonstrate that increasing the number of hydroxyethyl groups leads to a reduction in CMC.Thermodynamic analysis confirmed the spontaneous nature of micelle formation across all systems. Additionally, the analysis of limiting molar conductivity *Λ*_0_, and ion association constant, *K*_A_ further supports the strengthening of SAIL–drug interactions with increasing drug concentration, suggesting a significant role of molecular interactions in influencing micellization behavior. The observed trends highlight the potential of SAILs in pharmaceutical applications, particularly in enhancing the solubilization and delivery of hydrophobic drugs. Surface tension measurements revealed that increasing drug concentrations led to a reduction in both CMC and surface tension, indicating enhanced surface activity. The maximum surface excess concentration (*Γ*_max_) exhibited a direct correlation with drug concentration, while the introduction of hydroxyethyl groups in SAILs promoted bulk solubility, thereby reducing interfacial adsorption. Overall, these results provide valuable insights into the interplay between molecular structure, interfacial behavior, and thermodynamic stability of SAILs in pharmaceutical solutions, contributing to a deeper understanding of their potential applications in drug delivery and formulation.

The COSMO-derived *σ*-profiles results revealed that SAILs exhibit broader charge distribution, suggesting a greater potential for electrostatic interactions and enhanced solubility in polar environments, as inferred from the broader surface charge distribution, whereas aspirin showed more localized electron density due to their rigid aromatic structures. These findings underscore the critical role of charge distribution and molecular architecture in determining the physicochemical behavior of SAILs, highlighting their superior solvation properties and potential for micellar applications in pharmaceutical and colloidal systems.

## Future work

Future investigations should aim to elucidate the underlying mechanisms governing the interactions between surface-active ionic liquids (SAILs) and aspirin through comprehensive in vitro and in vivo studies. In vitro approaches may utilize advanced analytical techniques such as spectroscopy and microscopy to characterize the formation of SAIL– aspirin complexes and evaluate their effects on the drug’s solubility, permeability, and physicochemical stability. Concurrently, in vivo studies, including those employing appropriate animal models, could be employed to assess the pharmacokinetic profiles, therapeutic efficacy, and safety of SAIL-based delivery systems for aspirin. Furthermore, the initiation of clinical trials may offer valuable insights into the translational potential of these formulations, thereby facilitating the development of innovative drug delivery platforms for aspirin and other compounds with limited aqueous solubility. Expanding research efforts in these areas holds promise for enhancing the bioavailability, therapeutic outcomes, and patient adherence associated with aspirin therapy.

## Supplementary Information

Below is the link to the electronic supplementary material.

Supplementary Material 1

## Data Availability

The authors confirm that the data supporting the findings of this study are available within the manuscript, figures, tables, and supporting information files.
