# Supplementary material for: The surface and micellar properties of ethanolamine based surface active ionic liquids in the presence of drug aspirin
Source: Sci Rep. 2025 Oct 22;15:36936. doi: 10.1038/s41598-025-20946-2 (PMC12546906; doi:10.1038/s41598-025-20946-2)
Supplement: Supplementary file 1 — Supplementary Material 1 [file 41598_2025_20946_MOESM1_ESM.docx]

**Supporting Information**

**The surface and micellar properties of ethanolamine based surface active ionic liquids in the presence of drug aspirin**

**Elaheh Janbezar ^a^, Hemayat Shekaari^[[1]](#footnote-1)^* ^a^,, Shima Ghasemzadeh***^a^***, Mohammad Bagheri Hokm Abad***^a^*

*^a^Department of Physical Chemistry, Faculty of Chemistry, University of Tabriz, Tabriz, 5166616471, Iran*

* *Corresponding author. Tel.: +*984133393094. Fax: +984133340191.

E-mail address: [hemayatt@yahoo.com](mailto:hemayatt@yahoo.com) (H. Shekaari).

*
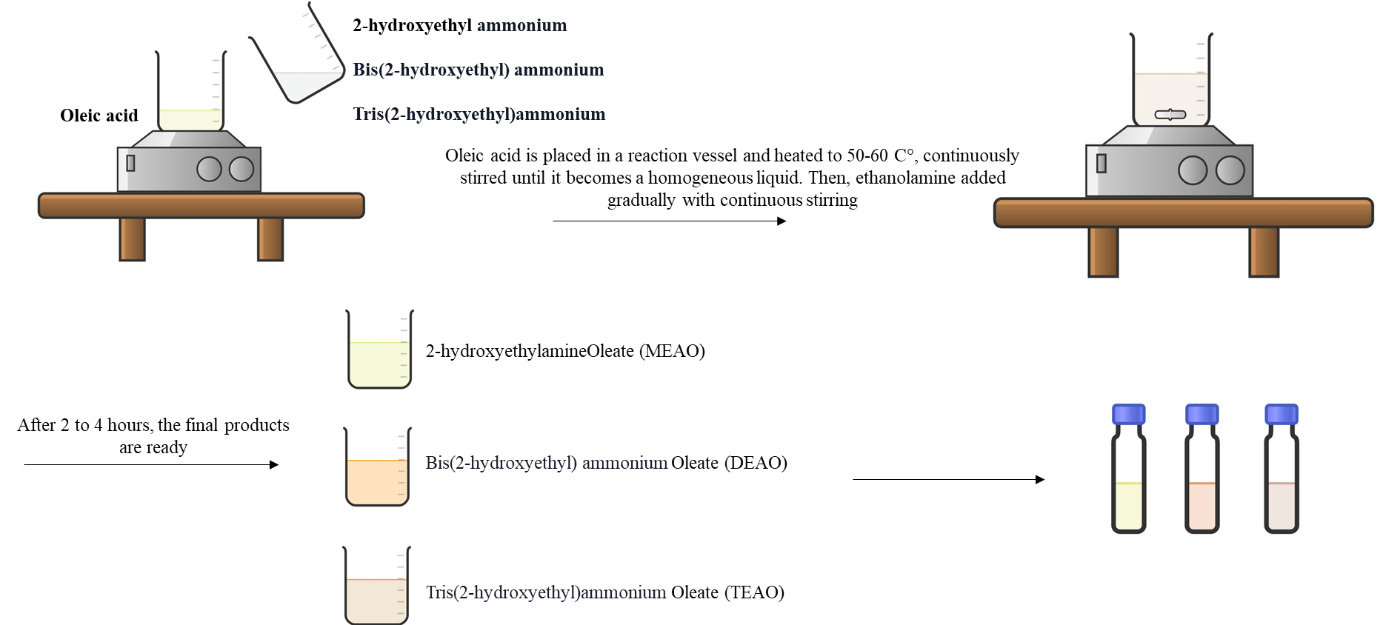
*

**Fig S1. Schematic of the prepration of the SAILs**


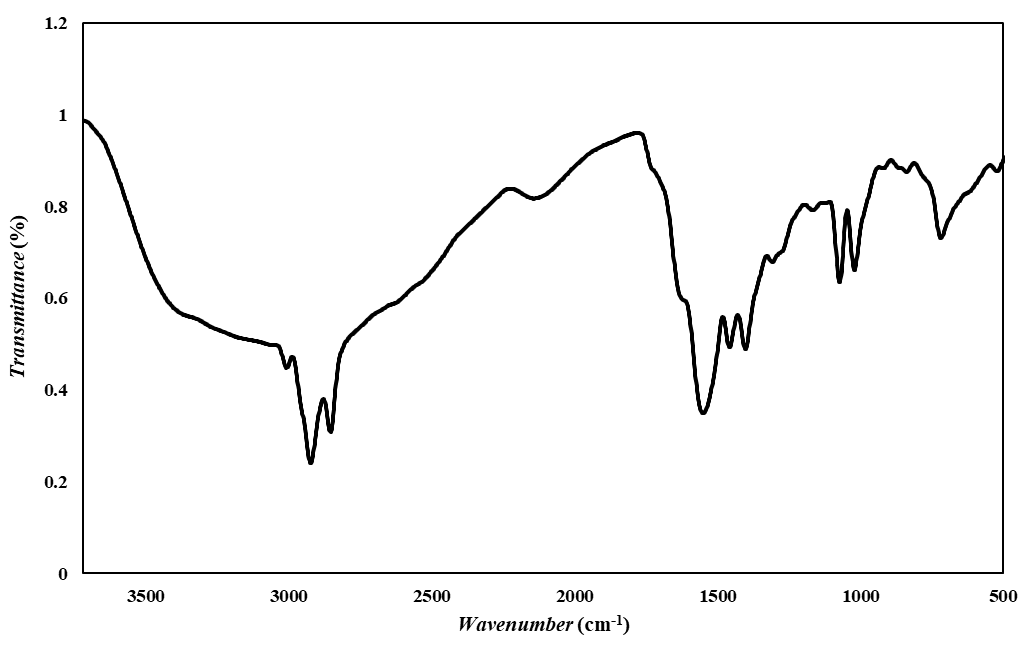


**Fig S2.** The FT-IR spectrum of [2-HEA][Ole].


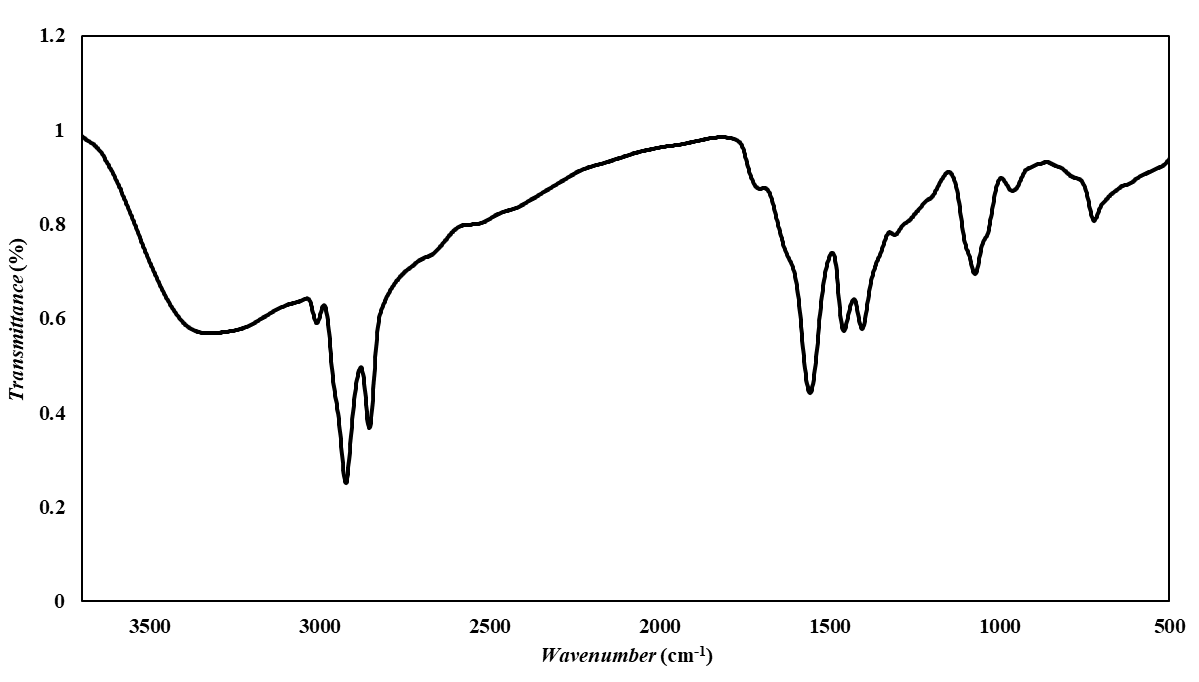


**Fig S3.** The FT-IR spectrum of the [BHEA][Ole].

**
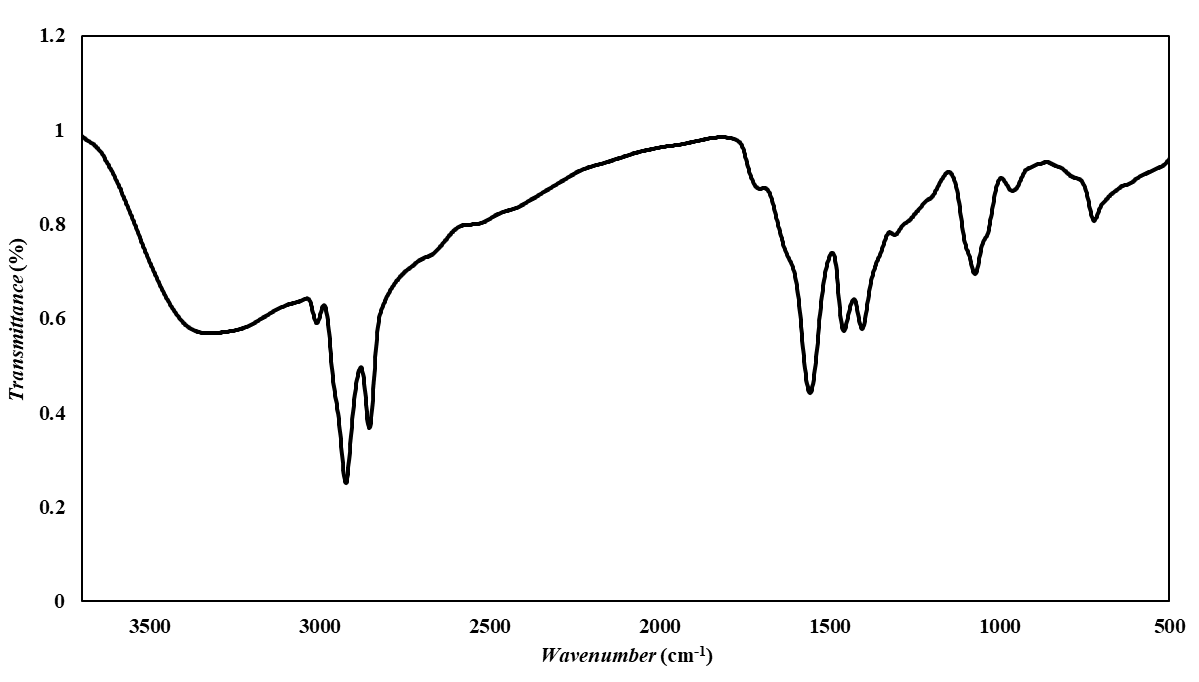
**

**Fig S4.** The FT-IR spectrum of [THEA][Ole].


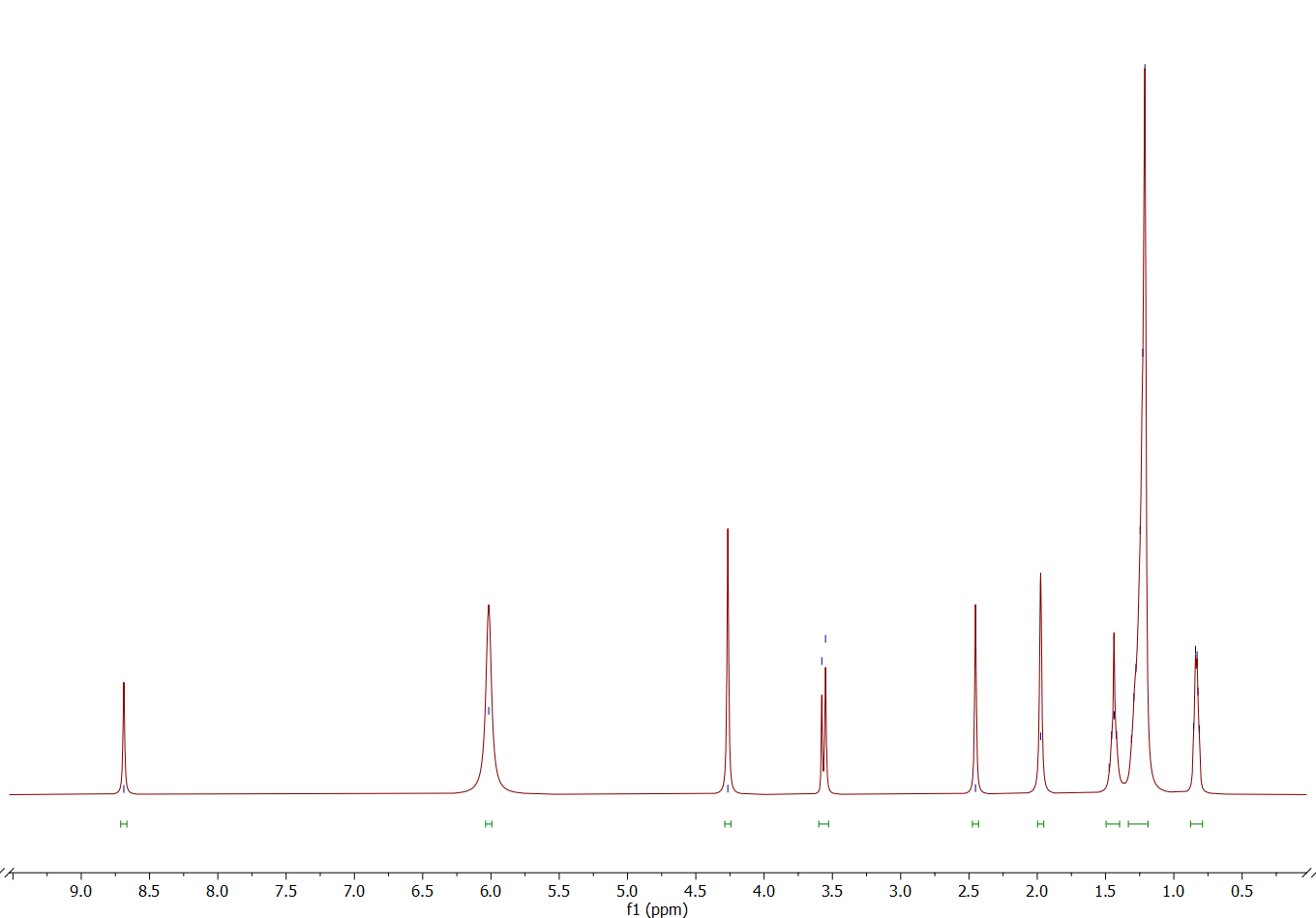


**Fig S5.** ^1^H-NMR spectrum (DMSO,400HZ) of [2-HEA] [Ole].

^1^H NMR (400 MHz, DMSO) δ 8.69 (s, 2H), 6.02 (s, 6H), 4.27 (s, 2H), 3.56 (d, J = 10.8 Hz, 2H), 2.45 (s, 2H), 1.98 (s, 2H), 1.44 (q, J = 7.1 Hz, 6H), 1.33 – 1.19 (m, 16H), 0.88 – 0.79 (m, 2H).


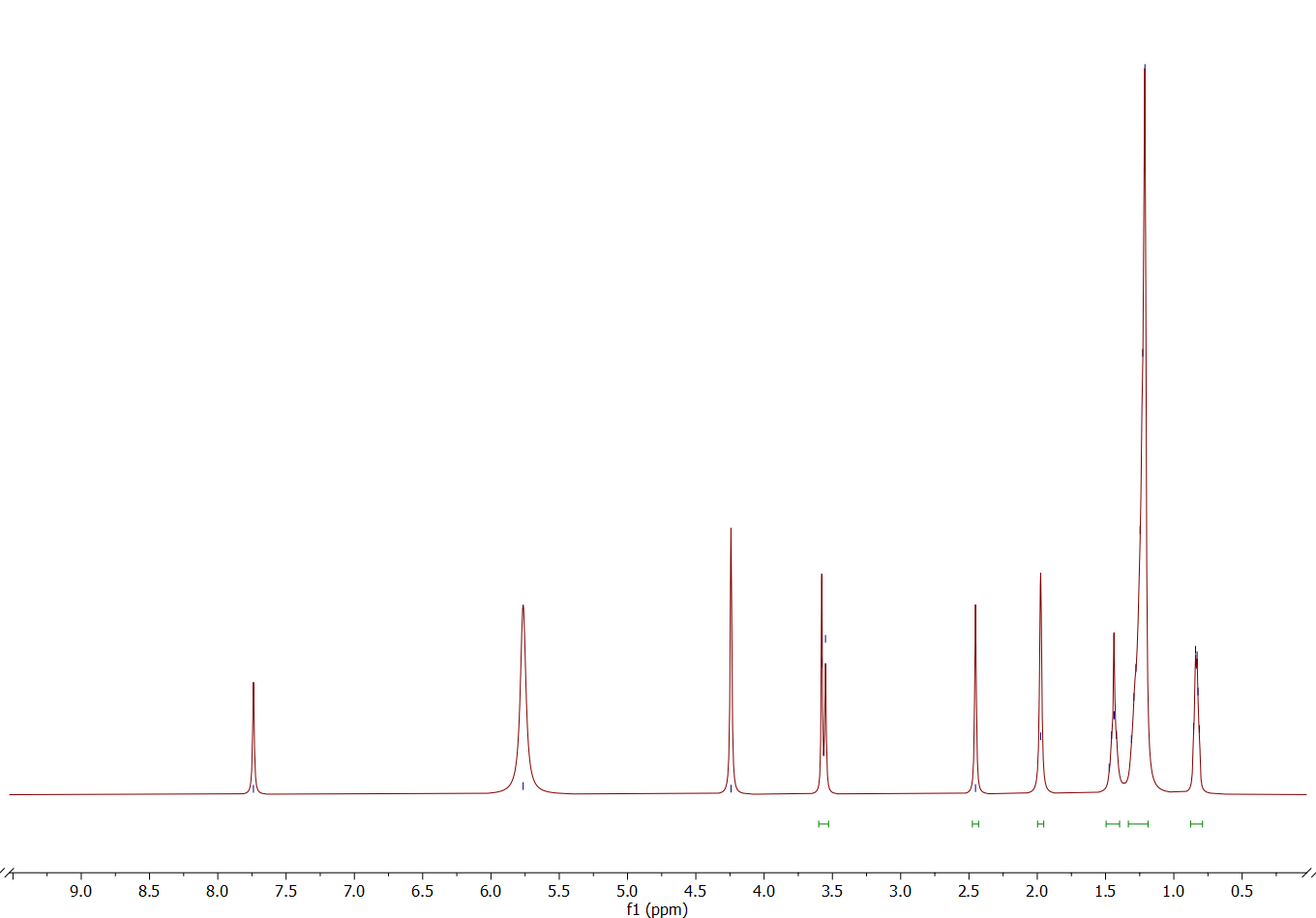


**Fig S6.** ^1^H-NMR spectrum (DMSO,400HZ) of [BHEA][Ole].

^1^H NMR (400 MHz, DMSO) δ 8.69 (s, 2H), 6.02 (s, 6H), 4.27 (s, 2H), 3.60 – 3.53 (m, 2H), 2.45 (s, 2H), 1.98 (s, 2H), 1.44 (q, J = 7.1 Hz, 4H), 1.33 – 1.19 (m, 18H), 0.88 – 0.79 (m, 4H).


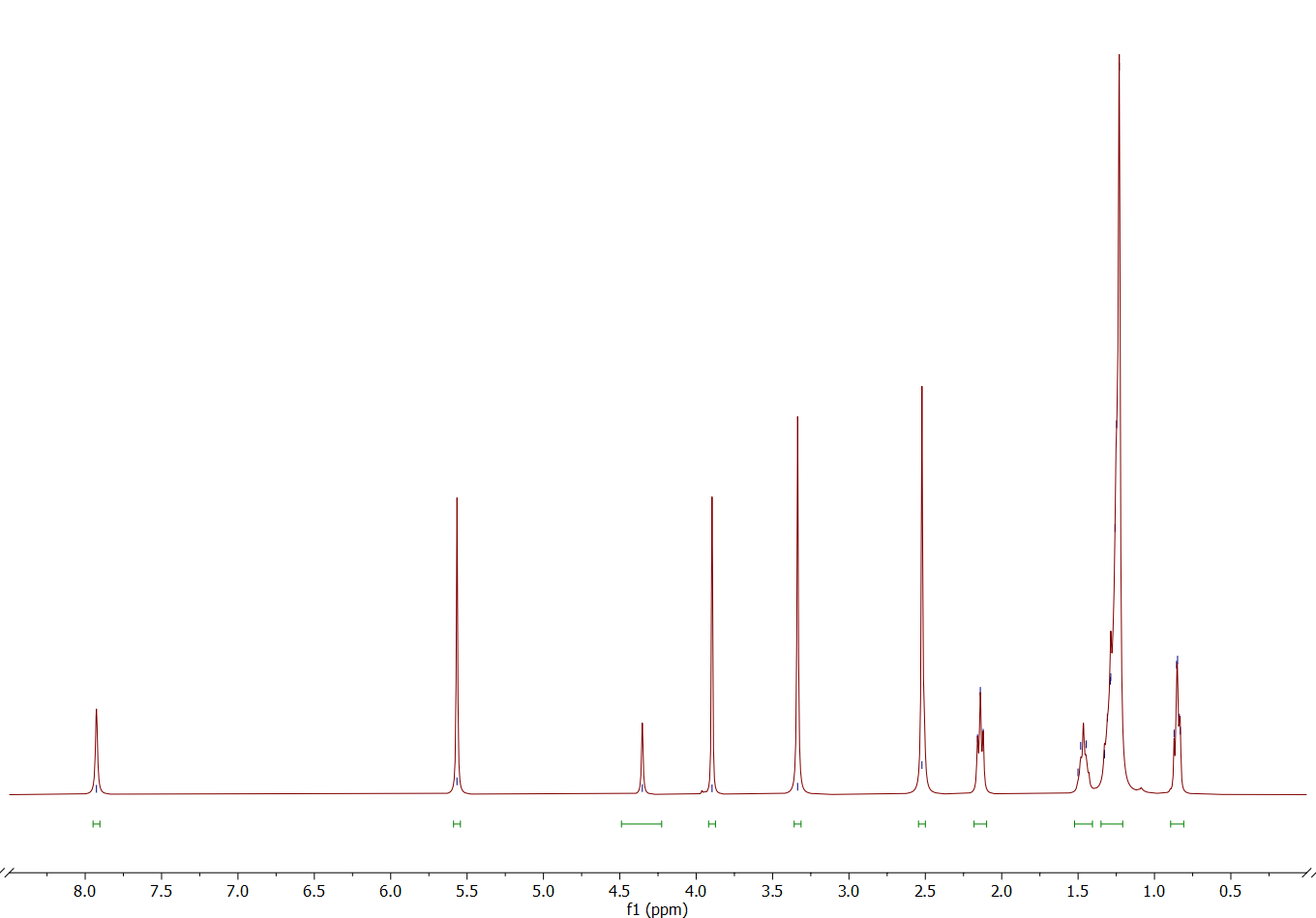


**Fig S7.** ^1^H-NMR spectrum (DMSO,400HZ) of [THEA][Ole].

^1^H NMR (400 MHz, DMSO) δ 7.93 (s, 2H), 5.57 (s, 3H), 4.35 (s, 1H), 3.90 (s, 2H), 3.34 (s, 5H), 2.52 (s, 5H), 2.14 (t, *J* = 7.4 Hz, 3H), 1.46 (q, *J* = 7.4 Hz, 3H), 1.35 – 1.21 (m, 24H), 0.85 (dt, *J* = 7.2, 4.5 Hz, 4H).


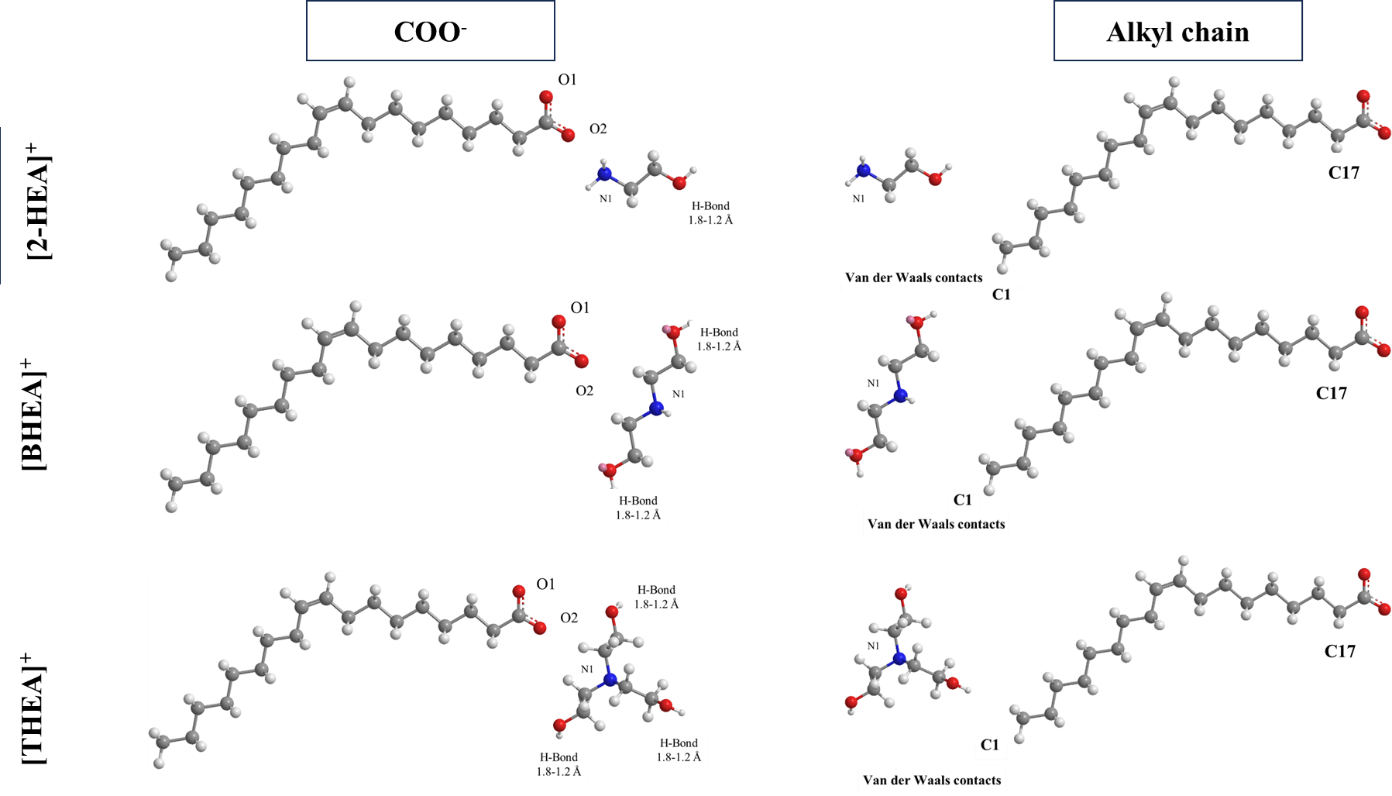


**Fig S8.** Optimized geometry of [2-HEA]^+^, [BHEA]^+^, and [THEA]^+^-oleate near [COO^-^] and near [Ole-], H-bond ~1.8–1.9 Å (COO^-^) and van der Waals contacts (alkyl chain), labels: N1, O1/O2 (COO^-^), and labels: N1, C1/C17 (alkyl chain).

1. [↑](#footnote-ref-1)
